# USP25 deubiquitinates cytosolic METTL3 to impede glioma proliferation via an m6A-independent pathway

**DOI:** 10.1016/j.isci.2025.113918

**Published:** 2025-10-31

**Authors:** Bingchang Zhang, Wanhong Han, Xin Gao, Wenhua Li, Jianhua Yu, Wujie Zhao, Jiawei He, Xiansheng Qiu, Zhenwei Lu, Liwei Zhou, Yahui Hu, Yuanyuan Xie, Yanyan Geng, Hanwen Lu, Wentao Zhao, Xinwen Liao, Shouren Chen, Xiyao Liu, Sifang Chen, Guowei Tan, Yaya Zhang, Zhanxiang Wang

**Affiliations:** 1 Department of Neurosurgery and Department of Neuroscience, Fujian Key Laboratory of Brain Tumors Diagnosis and Precision Treatment, Xiamen Key Laboratory of Brain Center, the First Affiliated Hospital of Xiamen University, School of Medicine, Xiamen University, Xiamen, Fujian 361102, China; 2 School of Basic Medical Science, Xiamen Medical College, Xiamen 361023, China; 3 School of Medicine, Xiamen University, Xiamen, Fujian 361102, China; 4 Xiamen Humanity Hospital, Xiamen, Fujian 361102, China; 5 Department of Neurosurgery and Department of Neuroscience, Fujian Key Laboratory of Brain Tumors Diagnosis and Precision Treatment, Xiamen Key Laboratory of Brain Center, the First Affiliated Hospital of Xiamen University, Fujian Medicine University, Fuzhou, Fujian 350122, China; 6 Clinical Research Institute of the First Affiliated Hospital of Xiamen University, Fujian Key Laboratory of Brain Tumors Diagnosis and Precision Treatment, Xiamen Key Laboratory of Brain Center, the First Affiliated Hospital of Xiamen University, School of Medicine, Xiamen University, Xiamen, Fujian 361102, China; 7 Xiamen Cell Therapy Research Center, Fujian Key Laboratory of Brain Tumors Diagnosis and Precision Treatment, the First Affiliated Hospital of Xiamen University, School of Medicine, Xiamen University, Xiamen, Fujian 361102, China; 8 Department of Laboratory Medicine, The First Affiliated Hospital of Xiamen University, School of Medicine, Xiamen University, Xiamen, Fujian 361003, China; 9 Department of Neurosurgery, Zhangzhou Affiliated Hospital of Fujian Medical University, 55 Shengli West Road, Zhangzhou, Fujian 363000, China; 10 Department of Medical Oncology, Fujian Key Laboratory of Brain Tumors Diagnosis and Precision Treatment, the First Affiliated Hospital of Xiamen University, School of Medicine, Xiamen University, Xiamen, Fujian 361102, China

**Keywords:** biological sciences, molecular biology, molecular mechanism of gene regulation, cancer

## Abstract

METTL3 exhibits distinct tumorigenic roles in the nucleus and cytoplasm, but whether its post-transcriptional modifications vary by subcellular localization remains unclear. Here, we identify METTL3 as a substrate of the deubiquitinase USP25, which stabilizes cytosolic METTL3 by cleaving its K48-linked polyubiquitin chains, preventing proteasomal degradation, without affecting nuclear METTL3 or global m6A abundance. The analysis of clinical glioma samples reveals a positive correlation between USP25 and METTL3 protein levels, with both significantly upregulated in high-grade glioma. Moreover, USP25 enhances EGFR expression through cytosolic METTL3, driving glioma progression. Our findings highlight that METTL3 undergoes distinct post-translational modifications based on its subcellular localization, providing new insights into the spatial regulation of METTL3 in gliomas.

## Highlights

• USP25 stabilizes METTL3 by cleaving K48-linked ubiquitin chains
• USP25-mediated stabilization of METTL3 does not affect global m6A abundance
• Cytosolic METTL3, stabilized by USP25, promotes glioma proliferation via EGFR
• USP25 and METTL3 are co-upregulated and correlated in high-grade glioma

## Graphical abstract

**Figure:**
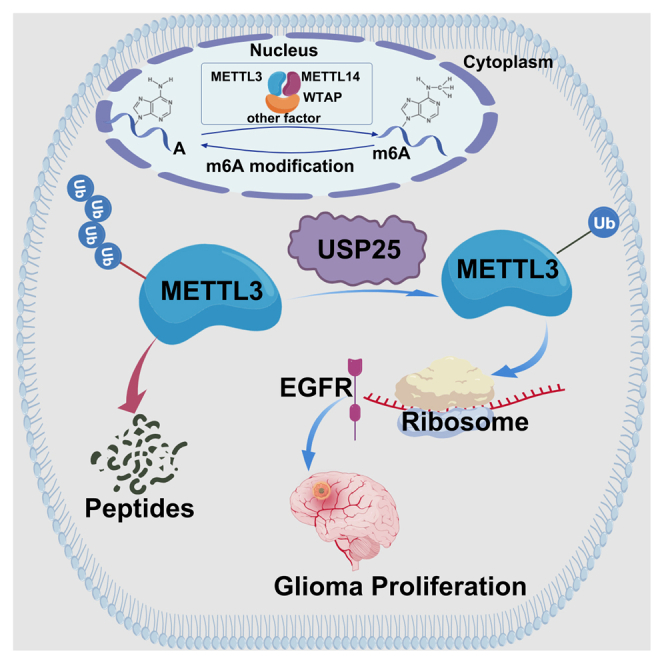

## Introduction

RNA methylation, particularly methylation at the N6 position of adenosine (m6A), has emerged as a critical epigenetic mechanism that regulates RNA stability, splicing, transport, and translation.^1^ M6A methylation is catalyzed by a multi-protein complex that includes METTL3, METTL14, and WTAP.^2^^,^^3^^,^^4^^,^^5^ As a core component of the m6A methyltransferase complex, METTL3 plays a pivotal role by transferring a methyl group to the N6 position of adenosine in RNA, resulting in the formation of m6A.^6^^,^^7^ Growing evidence suggests that METTL3 is also involved in biological processes beyond m6A modification.^8^^,^^9^ Moreover, the function of METTL3 is closely linked to its subcellular localization, highlighting the dynamic regulation of its activity in different cellular compartments. In the nucleus, METTL3 is primarily involved in functions related to m6A modification, while in the cytoplasm, it participates in functions unrelated to m6A modification.^9^^,^^10^^,^^11^

As a key regulator, METTL3 itself is tightly controlled by various posttranslational modifications (PTMs), including phosphorylation, ubiquitination, acetylation, and SUMOylation, which influence its protein stability, subcellular localization, and responsiveness to various cellular signals and stressors.^11^^,^^12^^,^^13^^,^^14^^,^^15^ For example, METTL3 acetylation promotes its translocation from the cytoplasm to the nucleus.^11^ Phosphorylation of METTL3 at the S43 site facilitates its relocation to DNA damage sites, where it participates in DNA repair.^16^ Additionally, phosphorylation of METTL3 by ERK enhances its binding to USP5, promoting its deubiquitination.^12^ However, it remains unclear whether METTL3 undergoes differential regulation in different cellular compartments.

Glioblastoma (GBM), characterized by its high heterogeneity and rapid clinical progression, is the most common and lethal primary brain tumor in adults.^17^^,^^18^^,^^19^ METTL3 has been extensively studied in the context of tumorigenesis.^10^^,^^11^^,^^14^^,^^20^ In our study, we found that METTL3 is significantly overexpressed in high grade glioma, METTL3 knockout inhibited the malignancy and tumorigenicity of GBM cells. Furthermore, we identified that METTL3 is regulated by USP25, a deubiquitinase belonging to the ubiquitin-specific protease (USP) family of DUBs. USP25 deubiquitinates and stabilizes cytoplasmic METTL3, but not nuclear METTL3. These findings suggest a potential mechanism for the precise regulation of METTL3 through post-translational modifications at different subcellular levels.

## Results

### METTL3 promotes glioblastoma progression

**Figure 1:**
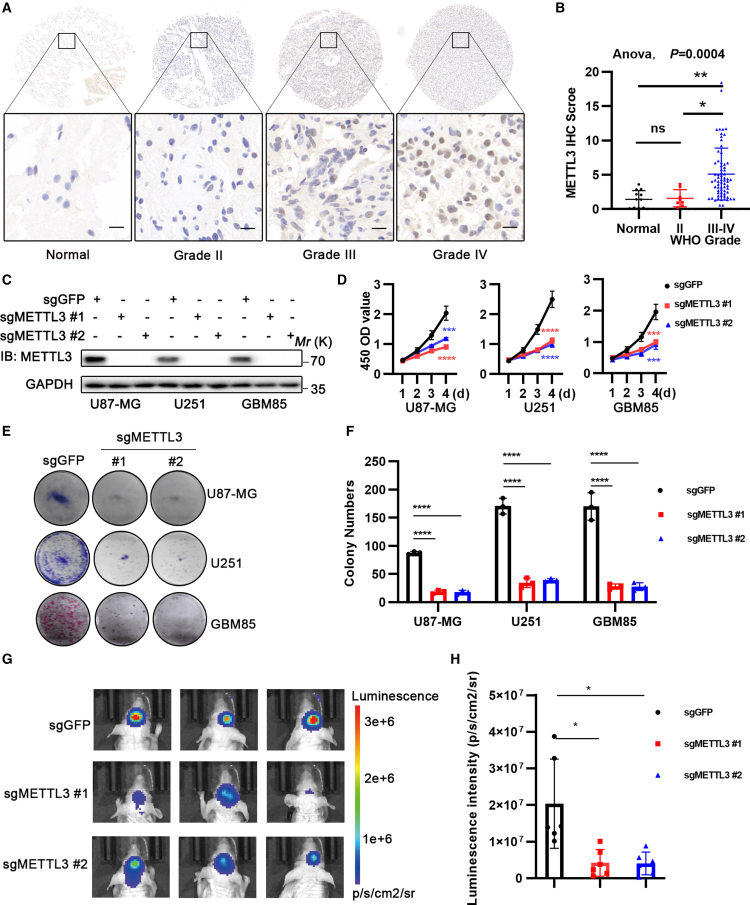
METTL3 promotes glioblastoma progression (A and B) IHC staining of METTL3 was conducted on sections from human glioma samples of grade II (*n* = 8), grade III (*n* = 14), grade IV (*n* = 54), and non-tumor brain tissue (*n* = 12) from a tissue microarray (TMA), with representative staining images provided. Scale bars: 100 μm for low magnification (10× 10, top panel) and 25 μm for high magnification (10× 40, bottom panel) (A), with quantitative analysis (B). (C) METTL3 was knocked out in U87-MG cells, U251 cells, and primary glioma cells (GBM85) by infecting them with lentivirus encoding two independent sgRNAs (sgMETTL3 #1 and #2), with sgGFP as a control for 6 days. Western blot was performed to detect METTL3 expression after 6 days of infection. (D) Cell proliferation was measured using the CCK-8 assay (*n* = 4 biologically independent experiments). (E and F) Colony counting was conducted on the 12th day after seeding. (*n* = 3 biologically independent experiments). (G and H) Representative bioluminescent images of orthotopic U87-MG-Luc glioma-bearing nude mice at day 21 post-intracranial cell implantation for each group (G), with the quantification of luminescence levels (H). (*n* = 5 mice/group). Data are represented as mean ± S.E.M. (B) Statistical analyses using one-way ANOVA with Tukey’s multiple comparison post hoc test were performed on GraphPad Prism, where ∗*p* < 0.05, ∗∗*p* < 0.01, and ∗∗∗*p* < 0.001. (D, F, and H) Statistical analyses using unpaired *t* test were performed on GraphPad Prism, where ∗*p* < 0.05, ∗∗∗*p* < 0.001, and ∗∗∗∗*p* < 0.0001.

Aberrant expression of METTL3 has been extensively reported in human cancers.^11^^,^^15^^,^^20^ The CPTAC database reveals that METTL3 protein expression is elevated in various tumors compared to normal tissues, particularly in GBM (Figure S1). To verify the clinical implications of METTL3 in glioma, we performed immunohistochemistry (IHC) staining to assess METTL3 expression in a glioma tissue microarray (TMA) cohort, with specimens collected from our institution. The TMA data revealed that METTL3 protein levels were significantly elevated in high-grade gliomas (WHO grades III and IV) compared to low-grade gliomas (WHO grade II) and non-tumor brain tissue (Figures 1A and 1B). This suggests a potential correlation between METTL3 expression and glioma malignancy. Subsequently, we conducted colony formation and CCK8 assays to evaluate the oncogenic potential of METTL3 in glioma. METTL3 knockout significantly reduced the colony formation capacity and cell viability in glioblastoma cell lines (U87-MG, U251) and primary glioma cells (GBM85) (Figures 1C–1F). To examine the role of METTL3 in tumorigenesis *in vivo*, we injected U87-MG cells with METTL3-depleted or control cells, both stably expressing firefly luciferase, into the brains of nude mice. All xenografted mice developed brain tumors, which were clearly visualized using quantitative bioluminescence imaging. Notably, U87-MG cells with METTL3 knockout formed smaller tumors compared to the control cells (Figures 1G and 1H). In summary, METTL3 plays a pivotal role in promoting glioblastoma progression.

### USP25 binds to METTL3 and regulates its ubiquitination

**Figure 2:**
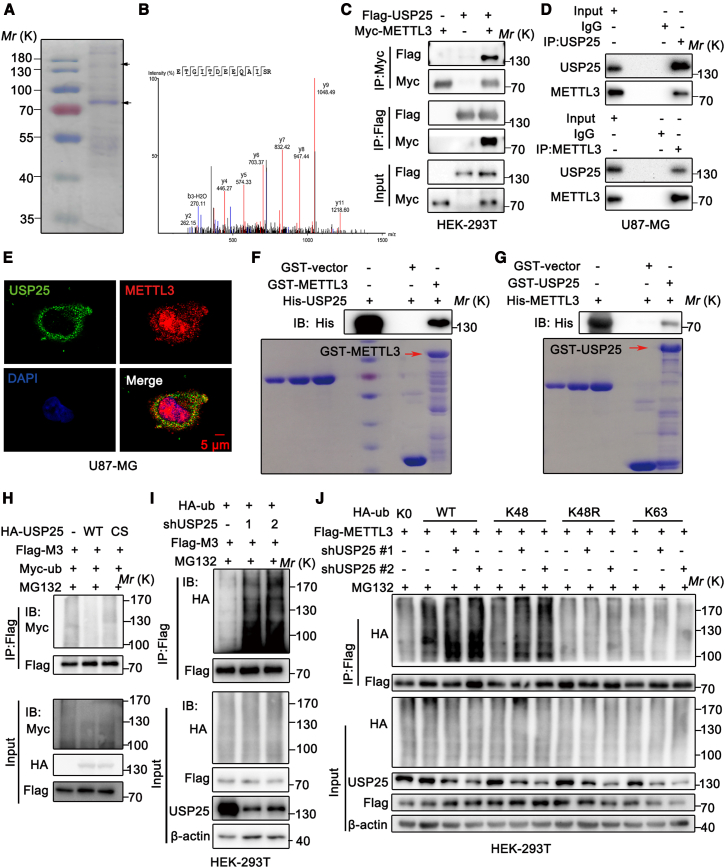
USP25 binds to METTL3 and regulates its ubiquitination (A and B) U87-MG cells were infected with lentivirus encoding Flag-METTL3 for 36 h, followed by stimulation with 25 μM MG132 for 6 h. Flag immunoprecipitation (IP) was performed, followed by SDS-PAGE and Coomassie blue staining, and the samples were analyzed by mass spectrometry. (C) HEK-293T cells were co-transfected with Flag-USP25 and Myc-METTL3, followed by IP using anti-Flag or anti-Myc affinity gel, respectively. (D) Endogenous METTL3 and USP25 in U87-MG cells were captured by IP using anti-METTL3 or anti-USP25 antibody, respectively. (E) Confocal laser scanning microscopy was used to observe the co-localization of METTL3 (red) and USP25 (green) in U87-MG cells. Nuclei were visualized with DAPI (blue). Scale bars = 5 μm. Colony counting was conducted on the 12th day after seeding. (*n* = 3 biologically independent experiments). (F and G) Purified His-USP25 or His-METTL3 was incubated with GST-USP25 or GST-METTL3 coupled to GSH-Sepharose, respectively. Proteins retained on Sepharose were then blotted with His antibodies. (H) HEK-293T cells were co-transfected with the indicated plasmids for 36 h, followed by stimulation with 25 μM MG132 for 6 h. Cell lysates were boiled and immunoprecipitated with anti-Flag affinity gel. Polyubiquitylated METTL3 was detected with anti-Myc antibody. (I) HEK-293T cells stably expressing control or USP25 shRNA were co-transfected Flag-METTL3 and HA-ub for 36 h, followed by stimulation with 25 μM MG132 for 6 h. Cell lysates were boiled and immunoprecipitated with anti-Flag affinity gel. Polyubiquitylated METTL3 was detected by anti-HA antibody. (J) HEK-293T cells stably expressing control or USP25 shRNA were co-transfected HA-tagged wild-type (WT) or indicated KR ubiquitin mutants with Flag-METTL3 for 36 h, followed by stimulation with 25 μM MG132 for 6 h. Cell lysates were boiled and immunoprecipitated with anti-Flag affinity gel. Polyubiquitylated METTL3 was detected by anti-HA antibody.

The GEPIA2 dataset indicated that the mRNA level of METTL3 was not differentially expressed in both GBM and LGG tissues compared to normal tissues (Figure S2A). The Cancer Genome Atlas (TCGA) database revealed a very low mutation rate, minimal frequency of METTL3 loss or gain, and negligible changes in its mRNA levels in both GBM and LGG (Figure S2B). It suggests that the dysregulation of METTL3 signaling primarily occurs through PTMs of this demethylase. METTL3 undergoes various PTMs, including phosphorylation, ubiquitination, lactylation, acetylation, and SUMOylation.^11^^,^^12^^,^^13^^,^^14^^,^^15^^,^^16^^,^^21^^,^^22^^,^^23^^,^^24^^,^^25^ To identify potential proteins involved in the degradation of METTL3, we performed protein mass spectrometry (MS) analysis. Flag-tagged METTL3 was isolated by immunoprecipitation (IP) and analyzed by MS (Figures 2A and 2B). The deubiquitinase USP25 was found to bind to METTL3. This interaction was further confirmed by co-immunoprecipitation (Co-IP) assays, where Flag-tagged USP25 and/or Myc-tagged METTL3 were co-transfected into HEK-293T cells (Figure 2C). To further validate the USP25-METTL3 interaction, reciprocal Co-IP assays were performed in U87-MG cells (Figure 2D) and U251 cells (Figure S3A), confirming that USP25 physically interacts with METTL3. Moreover, this interaction was also supported by immunofluorescence staining, which showed that endogenous METTL3 is largely co-localized with USP25 in the cytoplasm of both U87-MG cells (Figure 2E) and U251 cells (Figure S3B). To determine if the USP25-METTL3 interaction is direct, we generated and purified recombinant USP25 and METTL3. Purified GST-METTL3 or GST-USP25 interacted with His-USP25 or His-METTL3, respectively, under cell-free conditions, indicating a direct interaction between USP25 and METTL3 (Figures 2F and 2G). Mapping of the USP25 region required for METTL3 binding revealed that the USP domain (aa 166–718)^26^ of USP25 is both necessary and sufficient for the interaction with METTL3 (Figure S3C). Furthermore, His-USP25 was found to bind to the Gate-loop 1 (aa 368–424) and Interface loop (aa 425–500) of METTL3, which are part of the conserved methyltransferase domain (aa 368–580) of METTL3 (Figure S3D).^6^ Taken together, our results demonstrate that USP25 interacts with METTL3, and this interaction is mediated through the USP domain of USP25 and the C-terminal region of METTL3.

To further investigate whether USP25 functions as a bona fide deubiquitinase that deubiquitinates METTL3, we performed a deubiquitination assay. A significant reduction in polyubiquitylated METTL3 protein was observed in cells transfected with USP25^WT^, whereas the enzyme-inactive mutant (USP25^C178S^)^27^ was unable to decrease METTL3 ubiquitination (Figure 2H). To determine whether METTL3 is a direct deubiquitination substrate of USP25, we co-incubated polyubiquitylated METTL3 with purified Flag-USP25^WT^ or Flag-USP25^C178S^ under cell-free conditions. USP25^WT^, but not the USP25^C178S^ mutant, significantly reduced METTL3 ubiquitination *in vitro* (Figure S3E). Additionally, knockdown of USP25 by two specific short hairpin RNAs (shRNAs) in HEK-293T cells (Figure 2I) significantly increased METTL3 ubiquitination. When examining the linkage of METTL3 ubiquitination, we found that METTL3 was ubiquitinated through K48-specific chains (Figure S3F). Furthermore, we discovered that USP25 specifically regulates K48-linked, but not K63-linked, ubiquitin chains (Figure 2J). Taken together, our results suggest that USP25 is a bona fide DUB that targets METTL3 to remove its K48-linked polyubiquitinated chain.

### USP25 stabilizes METTL3

**Figure 3:**
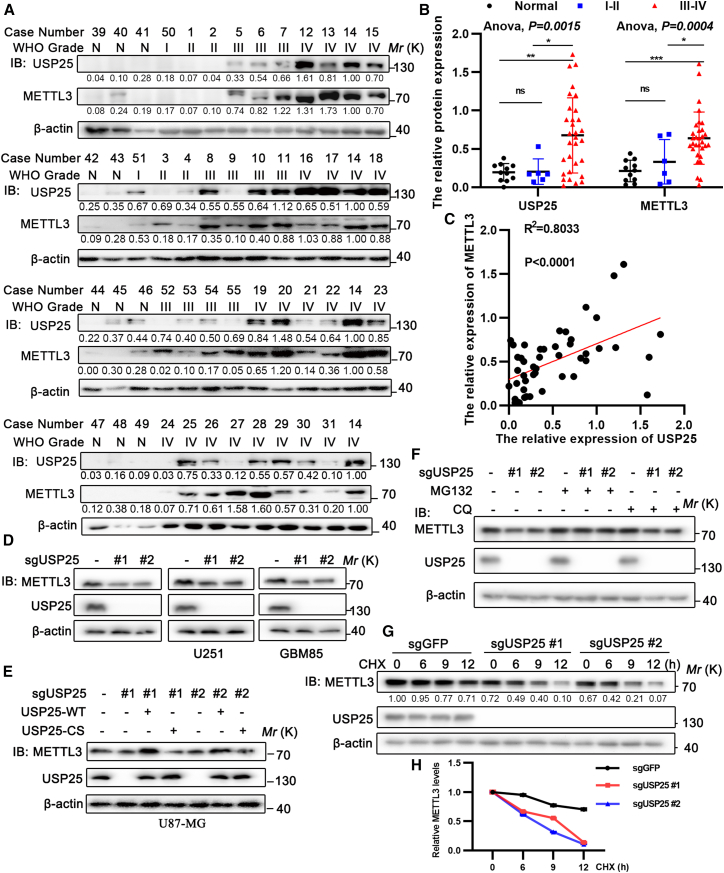
USP25 stabilizes METTL3 (A–C) The protein expression of METTL3 and USP25 in clinical samples was analyzed by Western blot (A), with optical density integrity (ODI) quantified using ImageJ (B), with Pearson’s correlation analysis of METTL3 and USP25 protein levels (C). N: non-tumor brain tissue, *n* = 11; WHO glioma grade I–II, *n* = 6, WHO glioma grade III–IV, *n* = 31. (D) Protein expression of METTL3 and USP25 in U87-MG, U251, and GBM85 cells infected with lentivirus encoding control or two individual USP25 sgRNAs was analyzed by Western blot. (E) Protein expression of METTL3 and USP25 in U87-MG and U251 cells infected with control or two individual USP25 sgRNAs, or USP25 sgRNAs together with sgRNA-resistant USP25^WT^ or USP25^C178S^, was analyzed by Western blot. (F) Protein expression of METTL3 and USP25 in U87-MG cells infected with control or two individual USP25 sgRNAs, and treated with or without 25 μM MG132 or 25 μM chloroquine (CQ) for 6 h, was analyzed by Western blot. (G and H) Protein expression of METTL3 and USP25 in U87-MG cells infected with control or two individual USP25 sgRNAs, and treated with or without 50 μg/μL cycloheximide (CHX) for the indicated time points (G).The intensity of METTL3, quantified using ImageJ, was normalized to β-actin (loading control) at each time point and then to the 0 h value (H). Data are represented as mean ± S.E.M. (B) Statistical analyses using one-way ANOVA with Tukey’s multiple comparison post hoc test were performed on GraphPad Prism, where ∗*p* < 0.05, ∗∗*p* < 0.01, and ∗∗∗*p* < 0.001.

Since USP25 is an ubiquitin-specific protease, and the tandem UIMs of USP25 preferentially bind to Lys48-linked ubiquitin chains,^28^ it is possible that USP25 may function to stabilize METTL3. To investigate the correlation between METTL3 and USP25, we analyzed the protein levels of USP25 and METTL3 in a broad panel of cancer cell lines and 37 clinical glioma samples, along with 11 brain tissues from patients with non-tumor (Figures 3A and S4A). The protein levels of both METTL3 and USP25 were higher in high-grade gliomas (WHO Grades III and IV) compared to low-grade gliomas and non-tumor brain samples (Figure 3B). Additionally, a significant positive correlation was observed between METTL3 and USP25 expression in both cell lines (*R*^2^ = 0.4193, *p* = 0.0228) (Figure S4B) and tissue samples (*R*^2^ = 0.8033, *p* < 0.0001) (Figure 3C). Next, knockout of USP25 using two specific CRISPR/Cas9 single guide RNAs (sgRNAs) led to a decrease in METTL3 protein levels, with no effect on METTL3 mRNA levels, in primary glioblastoma cells (GBM-85) and glioblastoma cell lines (U87-MG and U251) (Figures 3D and S4C). Similar results were obtained after knockdown of USP25 in various cancer cell lines, including breast cancer cell lines (MDA-MB-231 and T47D), non-small cell lung cancer (NSCLC) cell lines (A549 and H1299), and osteosarcoma cell lines (U2OS) (Figure S4D). Additionally, METTL3 protein levels were significantly decreased in USP25 homozygous-deficient (USP25^−/−^) MEFs and USP25 heterozygous-deficient (USP25^+/−^) MEFs compared to wild-type (USP25^+/+^) MEFs (Figure S4E). Reconstitution of USP25^WT^, but not USP25^C178S^, restored METTL3 protein levels in USP25 knockout U87-MG cells, U251 cells (Figure 3E), and USP25 knockdown MDA-MB-231 cells (Figure S4F), suggesting that USP25 upregulates METTL3 protein levels in an enzymatically dependent manner. Moreover, the decrease in METTL3 expression could be rescued by the addition of the proteasome inhibitor MG132, but not the autophagy inhibitor chloroquine (CQ) (Figures 3F, S4G, and S4H). Remarkably, USP25 knockout U87-MG cells and USP25 knockout U251 cells exhibited increased METTL3 protein degradation when treated with cycloheximide (CHX) (Figures 3G, 3H, and S4I). These results confirm that USP25 stabilizes METTL3 in a proteasome-dependent manner.

### Deubiquitination of METTL3 by USP25 does not affect its m6A RNA methyltransferase activity

**Figure 4:**
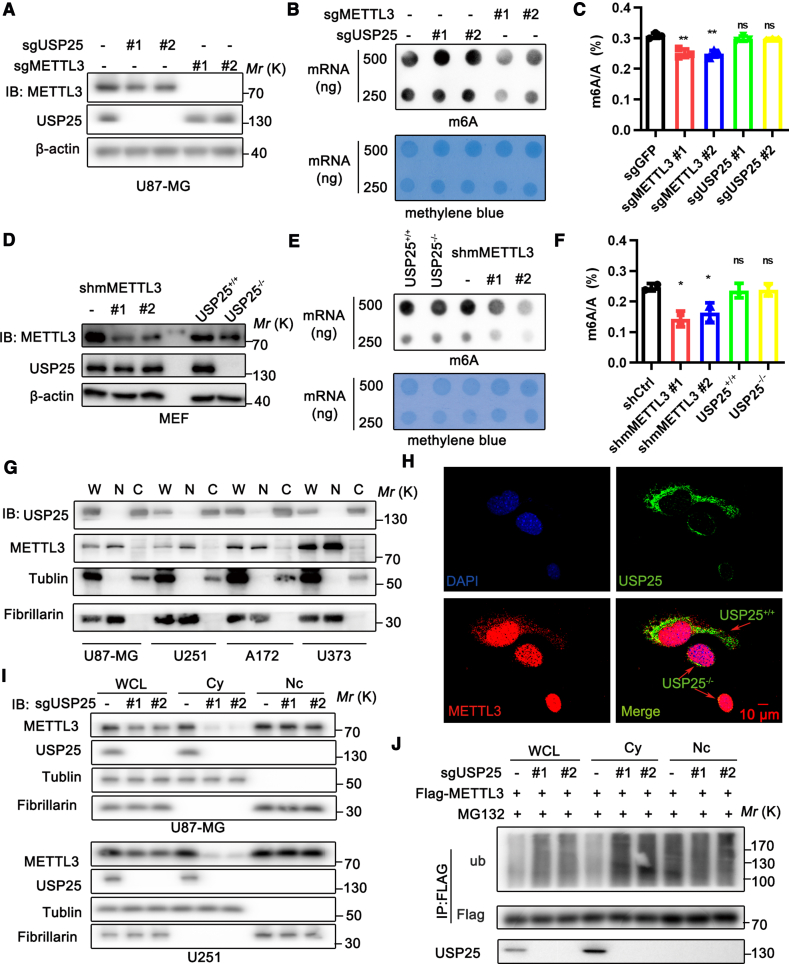
USP25 deubiquitinates and stabilizes cytoplasmic METTL3 (A–F) The protein levels of METTL3 and USP25 in indicated U87-MG cells and MEF cells were detected by Western blotting (A and D), with m6A levels in poly(A)+ RNA were detected by dot blot analysis, with methylene blue staining as a loading control (B and E), with the quantification of m6A/A ratios in poly(A)+ RNA were measured by LC-MS analysis (*n* = 3 biological replicates) (C and F). (G) Western blot analysis of the distribution of METTL3 and USP25 in nuclear and cytoplasmic fractions from U87-MG, U251, A172, and U373 cells. (H) Endogenous USP25 (green) and METTL3 (red) in USP25^+/+^ and USP25^−/−^ mixed MEF cells were detected by immunofluorescence staining. Nuclei were visualized with DAPI (blue). Scale bars, 10 μm. (I) Western blot analysis of METTL3 and USP25 distribution in nuclear and cytoplasmic fractions of U87-MG sgGFP control or sgMETTL3 (#1 and #2) cells. (J) Flag-METTL3 stably expressing U87-MG cells infected with lentivirus expressing sgGFP control or sgUSP25 (#1 and #2) were treated with MG-132 for 6 h. Total lysates, nuclear, and cytoplasmic fractions were immunoprecipitated with Flag antibody, and polyubiquitylated Flag-METTL3 was detected by anti-ubiquitin antibody. Data are represented as mean ± SEM. Statistical analyses using unpaired *t* test were performed on GraphPad Prism, where ∗*p* < 0.05 and ∗∗*p* < 0.01.

As the deubiquitination of METTL3 by USP25 affects its protein stability, we investigated whether deubiquitinated METTL3 alters its m6A RNA methyltransferase activity. To address this, we measured m6A modifications in mRNAs using dot blot and liquid chromatography-tandem mass spectrometry (LC-MS/MS). We observed a global decrease in m6A levels in METTL3 knockout U87-MG cells and METTL3 knockdown MEF cells (Figures 4A–4F). Interestingly, USP25 knockout U87-MG cells and USP25-depleted MEF cells showed no changes in m6A levels in mRNAs (Figures 4A–4F). As shown in Figure 2E, USP25 and METTL3 predominantly co-localized in the cytoplasm. We then performed nuclear/cytoplasmic fractionation followed by Western blot (WB) analysis to determine the subcellular localization. METTL3 was detected in both the cytoplasm and nucleus of glioblastoma cell lines U87-MG, U251, A172, and U373, whereas USP25 was almost exclusively detected in the cytoplasm (Figure 4G). Previous studies have suggested that cytoplasmic METTL3 may play roles other than m6A RNA methylation, which is primarily carried out by nuclear METTL3. This led us to hypothesize that USP25 affects the protein stability of cytoplasmic METTL3. To test this hypothesis, we mixed USP25^+/+^ and USP25^−/−^ MEF cells and performed immunofluorescence staining. We found that METTL3 was highly expressed in both the nucleus and cytoplasm of USP25^+/+^ MEF cells, but only in the nucleus of USP25^−/−^ MEF cells (Figure 4H). Moreover, knockout of USP25 specifically decreased the total and cytoplasmic levels of METTL3 without affecting nuclear METTL3 levels in U251 and U87-MG cells (Figure 4I). On the other hand, USP25 knockout in U87-MG cells led to increased cytoplasmic METTL3 ubiquitination (Figure 4J). In conclusion, our findings demonstrate that USP25 regulates the protein stability of METTL3 in the cytoplasm through deubiquitination, without affecting METTL3 stability in the nucleus or its m6A modification activity.

### USP25 enhances epidermal growth factor receptor expression through cytoplasmic METTL3, thereby promoting glioma proliferation

**Figure 5:**
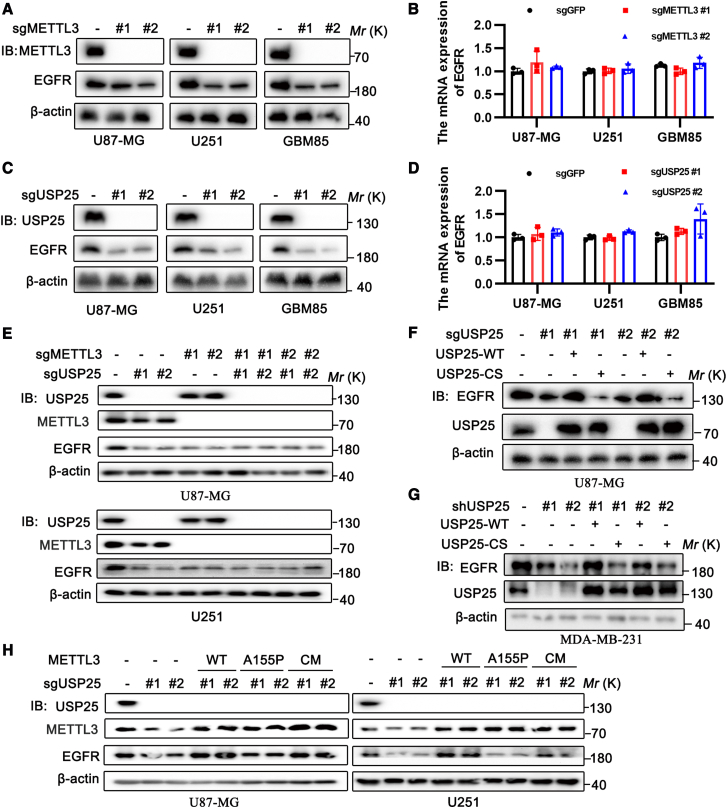
USP25 promotes EGFR expression through cytoplasmic METTL3 (A) The protein expression of EGFR in control and METTL3 knockout U87-MG, U251, and GBM85 cells was analyzed by Western blot. (B) The mRNA levels of EGFR in control and METTL3 knockout U87-MG, U251, and GBM85 cells were analyzed by qPCR. (C) The protein expression of EGFR in control and USP25 knockout U87-MG, U251, and GBM85 cells was analyzed by Western blot. (D) The mRNA levels of EGFR in control and USP25 knockout U87-MG, U251, and GBM85 cells were analyzed by qPCR. (E) The protein expression levels of EGFR from the USP25 and METTL3 individual or combined knockout U87-MG and U251 cells were analyzed by Western blot. (F) Protein expression of EGFR in U87-MG cells infected with control or two individual USP25 sgRNAs, or USP25 sgRNA together with sgRNA-resistant USP25^WT^ or USP25^C178S^, analyzed by Western blot. (G) Protein expression of EGFR in MDA-MB-231 cells infected with control or two individual USP25 shRNAs, or USP25 shRNA together with shRNA-resistant USP25^WT^ or USP25^C178S^, analyzed by Western blot. (H) Protein expression of EGFR in U87-MG and U251 cells infected with control or two individual USP25 sgRNAs, or USP25 sgRNA together with METTL3^WT^, METTL3A^155P^, or METTL3^CM^, analyzed by Western blot.

Emerging evidence has shown that METTL3 in the cytoplasm associates with ribosomes and contributes to translation via an m6A-independent mechanism.^20^ It has been reported that METTL3 enhances the translation of oncogenes such as epidermal growth factor receptor (EGFR), which is a key oncogene frequently amplified in glioblastomas. An early-phase study of CARv3-TEAM-E T cell therapy demonstrated significant tumor regression in patients with glioblastoma, supporting EGFR (including the EGFR variant III and wild-type EGFR) as a promising immunotherapeutic target in glioblastoma.^29^ Our experiments revealed that knocking out METTL3 in glioblastoma cell lines (U87-MG, U251) and primary glioma cells (GBM85) or knocking down METTL3 in non-small cell lung cancer (NSCLC) cell lines (A549, H1299) and breast cancer cell lines (MDA-MB-231, LM2) led to a reduction in EGFR protein levels without affecting its mRNA levels (Figures 5A, 5B, S5A, and S5B). Similar results were observed in USP25 knockout or knockdown cells (Figures 5C, 5D, S5C, and S5D). Furthermore, double knockout or knockdown of both USP25 and METTL3 did not further reduce EGFR protein levels, suggesting that USP25 and METTL3 function in the same pathway to regulate EGFR translation (Figures 5E and S5E). We next investigated whether restoring METTL3 activity could rescue EGFR translation in USP25-depleted cells. Overexpression of wild-type USP25, but not the USP25^C178S^ mutant, in USP25-depleted U87-MG cells and USP25 knockdown MDA-MB-231 cells, rescued EGFR protein expression (Figure 5F). Similarly, overexpression of wild-type METTL3 or the catalytic mutant METTL3^CM^ (aa395-398, DPPW/APPA)^30^ restored EGFR expression in USP25-depleted cells. However, the METTL3^A155P^ mutant,^10^ which cannot interact with eIF3h, failed to rescue EGFR expression (Figure 5H). These findings support the conclusion that USP25 enhances EGFR translation through cytoplasmic METTL3.

**Figure 6:**
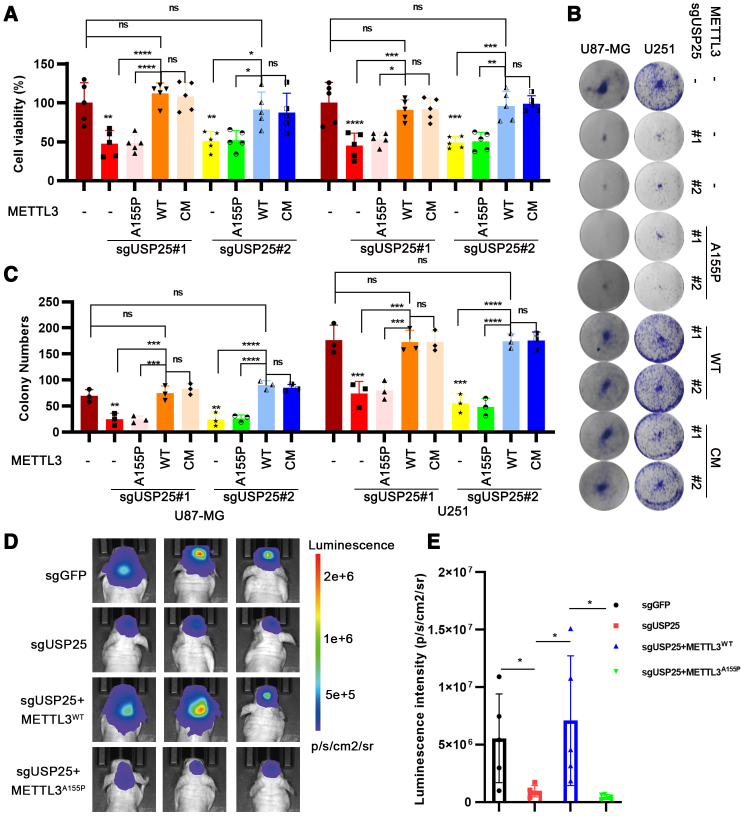
USP25-cytoplasmic METTL3-EGFR promotes glioma proliferation (A) Cell proliferation of the indicated U87-MG and U251 cells were measured using the CCK-8 assay (*n* = 4 biologically independent experiments). (B and C) Colony counting was conducted on the 12th day after seeding. (*n* = 3 biologically independent experiments). (D and E) Quantification of luminescence levels and representative bioluminescent images of orthotopic U87-MG-Luc glioma-bearing nude mice at day 21 post-intracranial cell implantation for each group. (*n* = 5 mice/group). Data are represented as mean ± SEM. Statistical analyses using unpaired *t* test were performed on GraphPad Prism, where ∗*p* < 0.05, ∗∗*p* < 0.01, ∗∗∗*p* < 0.001, and ∗∗∗∗*p* < 0.0001.

To explore the role of USP25 in cancer cell proliferation, we performed colony formation and CCK8 assays. USP25 knockout in U87-MG and U251 cells significantly reduced colony formation and cell viability, effects that could be rescued by overexpressing wild-type METTL3 or METTL3^CM^, but not METTL3^A155P^ (Figures 6A–6C). Subsequently, we conducted a mouse orthotopic tumor formation assay using sgGFP, sgUSP25, sgUSP25+METTL3^WT^, or sgUSP25+METTL3^A155P^ U87-MG-luc cells in nude mouse brains. Bioluminescence imaging showed that intracranial tumors generated from sgUSP25 cells were notably smaller compared to the control cells. Tumor growth was rescued by overexpressing wild-type METTL3, but not METTL3^A155P^ (Figures 6D and 6E). Our findings demonstrate that USP25 promotes glioma proliferation by stabilizing METTL3 in the cytoplasm, thereby enhancing EGFR translation.

## Discussion

METTL3 has emerged as a multifaceted protein that plays a pivotal role in regulating various aspects of RNA metabolism, including splicing, translation, stability, and decay.^1^ It exerts its effects through both m6A-dependent and m6A-independent mechanisms.^10^ METTL3 and the m6A modification are involved in various cancers through influencing various cancer-related cellular processes, like tumorigenesis, stemness of cancer cells, metastasis, and chemotherapy resistance.^31^^,^^32^ METTL3 or m6A modification holds promise as a therapeutic target in oncology. Glioma is the most common and aggressive form of brain cancer. METTL3 is upregulated in glioblastoma and associated with poor prognosis. The expression of METTL3 was upregulated in the TMZ-resistant GBM METTL3 knockdown promotes temozolomide sensitivity of glioblastoma cells.^33^^,^^34^ METTL3-mediated m6A modification promotes GSC proliferation, self-renewal, and tumorigenesis, promoting cancer stem cell maintenance and dedifferentiation of glioma cells.^35^^,^^36^ However, the specific mechanism by which METTL3 participates in the regulation of glioma remains to be further elucidated. Here, we found METTL3 is upregulated in high-grade glioma samples compared to non-tumor brain samples and low-grade glioma samples. METTL3 knockout significantly inhibited the proliferation of GBM cells both *in vitro* and *in vivo*.

METTL3 is subject to various PTMs. Phosphorylation of METTL3 by ERK at S43/S50/S525 enhances its stability by facilitating its interaction with USP5, which deubiquitinates and stabilizes METTL3, thereby increasing m6A methylation.^12^ Phosphorylation of METTL3 at S43 by ATM drives its localization to DNA damage sites, where it mediates the m6A methylation of DNA damage-associated RNAs, promoting homologous recombination-mediated repair of double-strand breaks (DSBs).^16^ Phosphorylation of METTL3 at S67 by TBK1 facilitates antiviral responses by regulating METTL3’s functions in both m6A modification and translation.^37^ METTL3 stabilization by PIN1, which prevented its ubiquitin-dependent proteasomal and lysosomal degradation, increased the m6A modification of transcriptional.^15^ Acetylation of METTL3 at K177 inhibits m6A modification and suppresses tumor metastasis by attenuating its translocation from the cytoplasm to the nucleus.^12^ Lactate drives the lactylation of METTL3 at K281 and K345, which was essential for capturing target RNAs and mediating RNA m6A modification.^14^ METTL3 undergoes ubiquitination by the E3 ligase STUB1, RNF113A, TRIM25, and deubiquitination by the deubiquitinating enzymes (DUBs) USP5, USP1/UAF1, and USP13.^12^^,^^23^^,^^38^^,^^39^^,^^40^^,^^41^ This modification can target METTL3 for proteasomal degradation.

Here, we found that the deubiquitinase USP25 is involved in the deubiquitination of METTL3 and affects its protein stability. However, USP25 does not affect the m6A levels in mRNAs. It is well-known that METTL3 contributes to cellular homeostasis through m6A-dependent and independent mechanisms. The multifaceted role of METTL3, in both m6A-dependent and m6A-independent manners, is primarily determined by its dynamic distribution in different cellular compartments. In the nucleus, METTL3, along with METTL14 and WTAP, catalyzes the m6A modification of pre-mRNAs, influencing their processing and function. Nuclear METTL3 also performs functions independent of its methyltransferase activity, often through interactions with transcription factors and chromatin modifiers. Cytoplasmic METTL3 allows it to influence the fate of mRNAs post-transcriptionally through m6A modifications. METTL3 also exerts non-m6A functions in the cytoplasm through direct protein-protein interactions and modulation of signaling pathways. Further investigation revealed that USP25 specifically regulates the ubiquitination of METTL3 in the cytoplasm, without affecting its ubiquitination in the nucleus. We also discovered that USP25, by modulating the ubiquitination of cytoplasmic METTL3, influences the translation of EGFR. This is the first time we have identified differences in the subcellular modification of METTL3, which may be related to the fact that USP25 is predominantly localized in the cytoplasm. Related studies have also suggested that nuclear METTL3 and cytoplasmic METTL3 have distinct functions in driving tumorigenesis and how tumor cells sense carcinogenic damage to coordinate the functions of METTL3 in these intracellular compartments. This study provides insights into the regulation of METTL3 at different subcellular levels.

USP25 is a deubiquitinating enzyme that, by cleaving ubiquitin from target proteins, plays an important role in regulating various physiological and pathological processes, including neurological disorders, inflammation, cancer, and metabolic regulation. USP25 is a multifunctional deubiquitinase that plays a significant role in tumor biology by modulating critical processes such as cell proliferation, survival, DNA repair, and metastasis. USP25 is upregulated in several types of cancer. USP25 is involved in stabilizing the HIF-1α transcription factor, which leads to metabolic reprogramming and supports tumor growth in pancreatic ductal adenocarcinoma (PDAC).^42^ USP25 plays a role in promoting non-homologous end-joining (NHEJ), a DNA repair pathway that contributes to chemoresistance in colon cancer cells. Inhibiting USP25 can increase the sensitivity of cancer cells to chemotherapy drugs such as IR, 5-Fu, and cisplatin.^43^ Here, we found USP25 is upregulated in high-grade glioma. USP25 knockout significantly inhibited the proliferation of GBM cells both *in vitro* and *in vivo*. USP25 could be a promising target for therapeutic intervention in cancer. Small-molecule inhibitors aimed at USP25 may provide a novel approach to cancer treatment. USP25/28 inhibitors CT1073 and CT1113 show broad anti-tumor activity. A preclinical study showed that CT1113 treatment demonstrated anti-leukemia efficacy in patient-derived T-ALL xenograft.^44^^,^^45^

Overall, we found that METTL3 is highly expressed in high-grade gliomas and plays a critical role in promoting glioma tumorigenesis. In the cytoplasm, METTL3 is regulated by the deubiquitinase USP25, which prevents its proteasomal degradation by cleaving K48-linked ubiquitin chains. This mechanism enhances the translation of EGFR, thereby sustaining tumor proliferation and progression. Our study underscores the pivotal role of METTL3 in glioma tumorigenesis and provides new insights into its post-translational regulation at the subcellular level.

### Limitations of the study

There are still several limitations to this study. First, due to the constraints of glioma surgery, which does not allow for extensive resection, we were unable to compare the expression levels of relevant proteins between tumor tissues and adjacent non-tumorous tissues. Second, most of the clinical glioma samples used in this study were collected shortly before the preparation of the tissue microarray, resulting in a lack of patient survival data. This limitation prevents us from assessing the correlation between METTL3 expression levels and patient survival outcomes. Additionally, the primary glioma cells we isolated were unable to form tumors in mice. Finally, this study did not investigate the inhibitory effects of targeted inhibitors on glioma, which will be a major focus of our future research.

## Resource availability

### Lead contact

Further information and requests for resources and reagents should be directed to and will be fulfilled by the lead contact, Zhanxiang Wang (wangzx@xmu.edu.cn).

### Materials availability

This study did not generate new unique materials or reagents.

### Data and code availability

• Original Western blot images, microscopic images/data reported here will be shared by the lead contact upon request.
• This paper does not report original code.
• Any additional information required to reanalyze the data reported in this paper is available from the lead contact upon request.

## Acknowledgments

This work was supported by grants from the 10.13039/501100001809National Natural Science Foundation of China (82072777), the Raman Medical Collaboration Project of The First Affiliated Hospital of Xiamen University (XFLM2023005), the 10.13039/501100003392Fujian Provincial Natural Science Foundation of China (2023J011624), the Fujian Provincial Clinical Research Center for Brain Diseases (2021FJSLCYX01), the Xiamen Clinical Research Center for Neurological Diseases (2021XMSLCYX01), and the Project of Xiamen Cell Therapy Research (3502Z20214001). We acknowledge all health care workers involved in the diagnosis and treatment of patients at the First Affiliated Hospital of Xiamen University and all the patients, supporters, and their families for their confidence in our work.

## Author contributions

B.Z. and Z.W. conceived the study. B.Z., Y.Z., and Z.W. designed the experiments and supervised the study. B.Z., W.H., X.G., W.L., W.Z., J.H., and X.Q. performed experiments. B.Z. and W.Z., X.L. analyzed data. X.G., W.L., J.Y., Z.L., L.Z., Y.H., S.C., X.L., and G.T. collected specimens. B.Z. and Z.W. wrote the paper. S.C., Y.X., Y.G., H.L., W.Z., S.C., X.L., G.T., and Y.Z. reviewed and edited the paper. All authors have read and approved the article.

## Declaration of interests

The authors declare no competing interests.

## STAR★Methods

### Key resources table

**Table:** 

REAGENT or RESOURCE	SOURCE	IDENTIFIER
**Antibodies**		

Anti-USP25 rabbit antibody	Abcam	Cat#ab187156
Anti-METTL3 mouse antibody	Abnova	Cat#B01P
Anti-USP25 mouse antibody	Santa cruz	Cat#sc-398414
Anti-METTL3 rabbit antibody	Proteintech	Cat#15073-1-AP
Anti-METTL14 rabbit antibody	Proteintech	Cat#26158-1-AP
Anti-EGFR rabbit antibody	Proteintech	Cat#18986-1-AP
Anti-Myc mouse antibody	Cell Signaling Technology	Cat#9B11
Anti-HA rabbit antibody	Roche	Cat#3F10
Anti-Flag rabbit antibody	Sigma Aldrich	Cat# F7425
Anti-GAPDH rabbit antibody	Proteintech	Cat#10494-1-AP
Anti-Actin rabbit antibody	Proteintech	Cat#60008
Anti-Tublin rabbit antibody	Proteintech	Cat#66240
Anti-Fibrillarin rabbit antibody	Proteintech	Cat#16021-1-AP
Anti-GFP rabbit antibody	Proteintech	Cat#66002-1-AP
Anti-GST rabbit antibody	Proteintech	Cat#14535-1-AP
Anti-His rabbit antibody	Proteintech	Cat#25940-1-AP
Anti-ubiquitin rabbit antibody	Santa cruz	Cat#sc-441120
Anti-m6A antibody	Synaptic Systems	Cat#202003
Anti-Flag M2 Affinity Gel	Sigma Aldrich	Cat#A2220
Anti-Myc Affinity Gel	Pierce	Cat#20168
Anti-HA Affinity Gel	Pierce	Cat#26181
Glutathione Sepharose	Pierce	Cat#16100
Anti-rabbit IgG-HRP antibody	Invitrogen	Cat#31460
Anti-mouse IgG-HRP antibody	Invitrogen	Cat#31430
Alexa Fluor® 488-labeled goat anti-mouse IgG	Invitrogen	Cat#A-11001
Alexa Fluor® 568-labeled Anti-Rabbit IgG	Invitrogen	Cat#A-11008

**Bacterial and virus strains**		

DH5a competent cells	This paper	N/A
Stbl3 competent cells	This paper	N/A
BL21 competent cells	This paper	N/A

**Biological samples**		

Human glioma tissue microarray	The First Affiliated Hospital of Xiamen University	N/A
Human glioma tissue	The First Affiliated Hospital of Xiamen University	N/A

**Chemicals, peptides, and recombinant proteins**		

Polybrene	Sigma Aldrich	Cat#H9268
Puromycin	Gibco	Cat#A1113803
DAPI	Sigma Aldrich	Cat#D9542
Cycloheximide	Sigma Aldrich	Cat# 239763-M
MG132	Sigma Aldrich	Cat#M8699
Chloroquine	Sigma Aldrich	Cat#C6628

**Critical commercial assays**		

RevertAid Reverse Transcriptase	Thermo	Cat#EP0442
SYBR Green Master	Roche	Cat#4913914001
Dynabeads mRNA Purification Kit	Invitrogen	Cat#
TRIzol reagents	Invitrogen	Cat#15596018
RiboMinus transcriptome isolation kit	Invitrogen	Cat# K155003

**Experimental models: Cell lines**		

Human: U87 MG	ATCC	HTB-14
Human: U251	ATCC	09063001
Human: HEK-293T	ATCC	CRL-1573
Human: A172	ATCC	CRL-1620
Human: U373	ATCC	HTB-17
Human: MDA-MB-231	ATCC	HTB-26
Human: LM2	ATCC	HTB-204
Human: T47D	ATCC	HTB-133
Human: A549	ATCC	CCL-185
Human: H1299	ATCC	CRL-5803
Human: U2OS	ATCC	HTB-96
Human: GBM85	This paper	N/A
Mouse embryonic fibroblast	This paper	N/A

**Experimental models: Organisms/strains**		

BALB/c-Nude mice	GemPharmatech	StrainNO.D000521

**Oligonucleotides**		

Human USP25 shRNA_1	GGAGACAACTTACTACCAAAC	N/A
Human USP25 shRNA_2	GCATTGGAATTTGCCTCAAGT	N/A
Human USP25 sgRNA_1	CAAGGCTTGCTGTAGTATCT	N/A
Human USP25 sgRNA_2	TGGAAACTTGGAATTAGCAG	N/A
Human METTL3 shRNA_1	GGGCCCAAGTGCAAGAATTCT	N/A
Human METTL3 shRNA_2	GCTCAACATACCCGTACTACA	N/A
Mouse METTL3 shRNA_1	GCACACTGATGAATCTTTAGG	N/A
Mouse METTL3 shRNA_2	GCACCCGCAAGATTGAGTTAT	N/A
Human METTL3 sgRNA_1	TTTGCCAGTTCGTTAGTCTC	N/A
Human METTL3 sgRNA_2	GTTGAAAAATTTCGCTCTCG	N/A
Human METTL3 qPCR primers	F: TCTGGGGGTATGAACGGGTA R: CTGGTTGAAGCCTTGGGGAT	N/A
Mouse METTL3 qPCR primers	F: ATCTTGGCTCTATCCGGCTG R: GATAGAGCTCCACGTGTCCG	N/A
Human EGFR qPCR primers	F: TCTGAGTGCAACCAGCAACA R: GTGGGGTCTGAGCTGTATCG	N/A
Human GAPDH qPCR primers	F: CCTGACCTGCCGTCTAGAAA R: CCCTGTTGCTGTAGCCAAAT	N/A
Mouse GAPDH qPCR primers	F: AAATGGGGTGAGGCCGGTGC R: ATCGGCAGAAGGGGCGGAGA	N/A

**Recombinant DNA**		

pLVX-puro-Flag-USP25	This paper	N/A
pLVX-puro-Flag-USP25 C178S	This paper	N/A
pLVX-puro-Flag-METTL3	This paper	N/A
pLVX-puro-Flag-METTL3 CM	This paper	N/A
pLVX-puro-Flag-METTL3 A155P	This paper	N/A
pcDNA3.1-Myc-METTL3	This paper	N/A
pcDNA3.1-HA-USP25	This paper	N/A
pcDNA3.1-HA -USP25 C178S	This paper	N/A
pGEX-4T-1-GST-METTL3	This paper	N/A
pGEX-4T-1-GST-METTL3-1-368	This paper	N/A
pGEX-4T-1-GST-METTL3-369-580	This paper	N/A
pGEX-4T-1-GST-METTL3-1-424	This paper	N/A
pGEX-4T-1-GST-METTL3-1-500	This paper	N/A
pGEX-4T-1-GST-METTL3-425-580	This paper	N/A
pGEX-4T-1-GST-METTL3-500-580	This paper	N/A
pGEX-4T-1-GST-METTL3-Δ (369–424)	This paper	N/A
pGEX-4T-1-GST-METTL3-369-424	This paper	N/A
pGEX-4T-1-GST-METTL3-Δ (369–500)	This paper	N/A
pGEX-4T-1-GST-METTL3-369-500	This paper	N/A
pGEX-4T-1-GST-METTL3-Δ (425–500)	This paper	N/A
pGEX-4T-1-GST-METTL3-425-500	This paper	N/A
pGEX-4T-1-GST-USP25	This paper	N/A
pGEX-4T-1-GST-USP25-1-142	This paper	N/A
pGEX-4T-1-GST-USP25-166-718	This paper	N/A
pGEX-4T-1-GST-USP25-166-1055	This paper	N/A
pGEX-4T-1-His-METTL3	This paper	N/A
pGEX-4T-1-His-USP25	This paper	N/A

**Software and algorithms**		

Step One Plus Real-Time PCR System	Applied Biosystems	https://www.thermofisher.cn/
SlideViewer2.6	3DHISTECH	https://www.3dhistech.com/
the AniView100Pro multimodal *in vivo* imaging system	Guangzhou Biolight Biotechnology	https://www.blt-imaging.com/
GraphPad Prism 8	Graphpad	https://www.graphpad.com/
Peaks Studio X software	Bioinformatics Solutions lnc.	https://www.bioinfor.com/peaks-studio-x-plus/

### Experimental model and study participant details

#### Clinical sample collection and tissue microarray (TMA) and immunohistochemistry

TMA samples were extracted from patients designated for surgical resection within the Neurosurgery Department of the First Affiliated Hospital of Xiamen University between September 2019 and April 2024. Histopathologists performed pathological confirmation of the diagnosis according to the WHO criteria. All patients or relatives provided written informed consent. Such an investigation complied with the Declaration of Helsinki and the Ethics Committee of the First Affiliated Hospital of Xiamen University approvals. The TMA containing 38 glioma cases (grade II (*n* = 4), grade III (*n* = 7), grade IV (*n* = 27)), 6 normal brain tissues with duplicate cores per case. IHC staining of METTL3 in TMA was conducted by Servicebio Technology (Wuhan, China).

#### Cell culture and cell lines

Human glioblastoma cell lines (U87-MG, U251, A172, U373), breast cancer cell lines (MDA-MB-231, LM2, T47D), lung cancer cell lines (A549, H1299), HEK-293T cells, and mouse embryonic fibroblast (MEF) cells were cultured with DMEM supplemented with 10% fetal bovine serum (FBS) and antibiotics. Human osteosarcoma cell line U2OS were cultured in McCoy’s 5a medium with 10% FBS and antibiotics, and cells were grown in a 5% CO2 cell culture incubator at 37°C. Human primary glioblastoma cells isolation and culture were performed according to the method described as previously.^46^

#### Plasmid construction, lentiviral production and infection

Human METTL3 or USP25 fragment were PCR-amplified and cloned into the pcDNA3.1 vector, the pLVX-IRES-Puro-Flag lentiviral vector, the pGEX-4T-1-GST vector or pET-21b-His vector. Point mutation and deletion mutants for METTL3 and USP25 were generated by KOD-Plus-Neo DNA polymerase (Toyobo, KOD-201) as QuikChange Mutagenesis Kit Site-Directed described47 and verified by sequencing. The shRNA targeting the human/mouse METTL3 and human USP25 were cloned into the pLV-H1-EF1α-puromycin lentiviral vector. The sgRNA targeting the human *USP25* and *METTL3* were cloned into the lentiCRISPR v2 vector. METTL3/USP25 shRNA/sgRNA resistance plasmids were generated by introducing synonymous mutations into the shRNA/sgRNA targeting sequence using the KOD-Plus-Neo DNA polymerase. The lentiviral vector and the packaging cassettes, pMDL, REV, and VSVG, were cotransfected into HEK-293T cells. Viruses were collected and filter it through a 0.45 μm filter unit at 48 h after transfection and then used to infect cells with Polybrene (8 mg/mL, Sigma); 48 h after infection, puromycin was added to the culture medium to select the infected cells.^47^

#### Co-immunoprecipitation and western blot

Cell was collected and lysed using IP lysis buffer (Thermo Fisher Scientific) then the supernatant was incubated with specific antibodies and protein A/G agarose beads with rotating at 4°C for overnight. The Gel were then washed with cold lysis buffer for six times on ice, resuspended in 2 × SDS loading buffer and separated by SDS-PAGE, followed by immunoblotting with the specified antibodies.

#### Protein expression, purification and GST pull-down assay

Plasmids expressing the recombinant proteins were transformed into BL21 Escherichia coli and then the recombinant proteins were induced by IPTG at 18°C overnight. The bacteria were lysed in cold NETN buffer in the presence of the protease inhibitor mixture by sonication on ice. Then GST-tagged proteins were purified by glutathione Sepharose (Pierce, 16100) according to the manufacturer’s protocol. For GST pull down assay, about 10 μg of GST fusion proteins or GST control bound to glutathione Sepharose were incubated with recombinant His-tagged protein with rotating for 2 h at 4°C. The sepharoses were washed six times with NETN buffer on ice, resuspended in 30 μL of 2 × SDS loading buffer, and resolved on SDS–PAGE and detected by western blot analysis.

#### Protein LC-MS/MS analysis

After staining of gels with Coomassie blue, excised gel segments were subjected to in-gel trypsin digestion, followed by dying. Samples were analyzed on a nanoElute (Bruker) coupled to a timsTOF Pro (Bruker) equipped with a CaptiveSpray source. Peptides were dissolved in 10 μL 0.1% formic acid and were auto-sampled directly onto a homemade C18 column (35 cm × 75 μm i.d., 1.9 μm 100 Å). Samples were then eluted for 60mins with linear gradients of 3–35% acetonitrile in 0.1% formic acid at a flow rate of 300 nL/min. Mass spectra data were acquired with a timsTOF Pro mass spectrometer (Bruker) operated in PASEF mode. The raw files were analyzed by Peaks Studio X software against uniprot database.

#### RNA isolation and RT–qPCR

Total RNA was isolated with TRIzol reagents (Invitrogen, 15596018) and cDNA was synthesized using RevertAid Reverse Transcriptase (Thermo, EP0442) according to the manufacturer’s instructions. RT–qPCR analyses were performed using SYBR Green Master (Roche, 4913914001) with the Step One Plus Real-Time PCR System (Applied Biosystems). The 2^−ΔCt^ method was used to quantitate fold changes by normalizing to GAPDH.

#### Immunofluorescence staining

Cells were seeded onto glass coverslips for the experiment. 24 h later, cells were then fixed with 4% formaldehyde for 10 min at room temperature and permeabilized with 0.1% Triton X-100 for 5 min, blocked with PBS plus 1% BSA for 1 h, incubated with anti-USP25(santa cruz, sc-398414, 1:200) and anti-METTL3 (Proteintech, 15073-1-ap, 1:200) antibody at 4°C overnight and then with incubated with Alexa Fluor 488-labeled goat anti-mouse IgG (Invitrogen, 1:200), Alexa Fluor 568-labeled Anti-Rabbit IgG (Invitrogen, 1:200), and DAPI in the dark for 30 min at room temperature. Localization of USP25 and METTL3 were visualized by confocal microscopy.

#### *In vivo* ubiquitination assay

Co-transfected with indicated plasmids HEK-293T cells were treated for 6h with 25 μM MG132 before being harvested. The cells were lysed in modified RIPA buffer (50 mM Tris-HCl (pH 8.0), 0.1% SDS, 1% Sodium deoxyohdine, 1% Triton X-100, 150 mM NaCl, 1 mM EDTA, 10 mM NaF, 1 mM Na3VO4, 1 mM PMSF, 1% EDTA-free, and 1 mM NEMI). Add SDS to a final concentration of 1% and boiled for 5 min, centrifuged to remove cell debris and diluted 10 times with modified RIPA buffer. The cell extracts were subjected to immunoprecipitation with the indicated antibodies, washed with ubiquitin wash buffer (50 mM Tris-HCl (pH 8.0), 0.1% Triton X-100, 140 mM NaCl, 1 mM EDTA) for six times on ice and separated by SDS–PAGE.^48^

#### *In vitro* deubiquitination assay

HA-ubiquitin and Flag-METTL3 were co-expressed in HEK-293T cells. After cells were treated with 10 mM MG132 for 6h, ubiquitinated Flag-METTL3 was isolated by IP with anti-Flag M2 Affinity Gel (SigmaAldrich). After washing with the ubiquitin wash buffer, the proteins were eluted by 3 X Flag peptide (SigmaAldrich). In a parallel experiment, Myc-USP25 (WT or C178S) or vector was expressed in HEK-293T cells, purified by IP with anti-Myc Affinity Gel (Pierce) and eluted with Myc peptide (Pierce), and subsequently incubated with ubiquitinated Flag-METTL3 in a deubiquitylation buffer (50 mM Tris-HCl (pH 8.0), 50 mM NaCl, 1 mM EDTA, 10 mM DTT, 5% glycerol) for 16 h at 37°C. The ubiquitinated status of METTL3 was analyzed by western blot with HA antibody.^48^

#### Cytoplasmic/nuclear protein extraction

Cells were harvested and resuspended with buffer A (10 mM HEPES (pH 7.9), 10 mM KCl, 0.1 mM EDTA,1 mM DTT, 1 mM PMSF, 1% EDTA-free) by vortexing and standed on ice for 5–10 min and then NP-40 was added to a final concentration of 2%(v/v) and vortexed for 10 s. The lysates were centrifuged, and the supernatant was transferred to a new tube and saved as cytoplasmic fraction. The nuclear pellet was resuspended and washed with buffer A twice and resuspended in buffer B (20 mM HEPES (pH 7.9), 400 mM NaCl, 1 mM EDTA, 1 mM DTT, 1 mM PMSF, 1% EDTA-free) and incubated on ice for 30 min. The lysates were centrifuged, and the supernatant was transferred to a new tube and saved as nuclear fraction.^11^

#### mRNA m6A quantification by LC-MS/MS and m6A dot blot

Total RNA was isolated with Trizol reagent (Invitrogen) and mRNA was enriched using Dynabeads mRNA Purification Kit (Invitrogen) according to the manufacturer’s instruction, followed by removal of contaminated rRNA with RiboMinus transcriptome isolation kit (Invitrogen, K155003). For the m6A dot blot assay, the nylon membrane was activated by methanol before spotting the RNA. mRNA was denatured at 72°C for 5 min and chilled on ice immediately. Then, 2 μL denatured RNA was spotted in duplicate on activated nylon membrane and ultraviolet cross-linking was conducted for 10 min. After cross-linking, one membrane was measured in 5% milk blocking buffer for 1 h at room temperature and then incubated with m6A antibody (Synaptic Systems, #202003) for 2 h at room temperature. Another identical membrane methylene blue stained as a loading control. For the m6A quantification by LC-MS/MS assay, the isolated mRNAs were subsequently digested into nucleosides by 1 U nuclease P1 (Sigma, 8630) in 25 μL buffer containing 25 mM NaCl, 2.5 mM ZnCl2 at 42°C for 2 h, followed by the addition of NH4HCO3 (1 M, 3 μL) and 1 U alkaline phosphatase (Sigma, 85L3R). After an additional incubation at 37°C for 2 h, the solution was centrifuged at 13000 rpm for 10 min at 4°C, and 5 μL of the solution was injected into LC-MS/MS. The nucleosides were quantified by using the nucleoside-to-base ion mass transitions of 285 to 153 (d3-m6A), 282 to 150 (m6A) and 268 to 136 (A). The total contents of m6A and A were quantified on the basis of the corresponding standard curves generated using pure standards, from which the m6A/A ratio was determined from the calculated concentrations.^49^

#### Colony formation assay

Cell was seeded into 12-well plates (*n* = 3), followed by cell culture for specific day. After crystal violet staining, the colony number was calculated for analysis.

#### Cell proliferation assay

Cells were seeded in 96-well plates (2 000 cells/well). Cell proliferation, represented by the OD value measured using a microplate reader at 450 nm, was studied at specific day using a CCK8 Kit (ApexBio, Houston, TX, USA), following the manufacturer’s protocol.

#### Intracranial xenograft model in nude mice and imaging

Intracranial xenograft model was performed according to the method described as described previously.^46^ Female BALB/c nude mice (6 weeks old) were used to establish the intracranial xenograft tumor model. The mice were randomly assigned into groups. After anesthesia, each mouse was secured in a stereotaxic apparatus. Using the bregma as the reference point, a burr hole was drilled at 1.2 mm lateral and 1.2 mm anterior to the bregma. A microsyringe was lowered to a depth of 3 mm below the dura mater. A total of 6 μL of PBS containing 2.5 × 10ˆ5 luciferase-expressing tumor cells was injected over 10 min, and the syringe was left in place for an additional 10 min to prevent reflux. One month after injection, tumor growth was monitored using the AniView100Pro multimodal *in vivo* imaging system (Guangzhou Biolight Biotechnology Co., Ltd, China) via bioluminescence imaging. Quantification of tumor signals was performed using AniView Pro software (Guangzhou Biolight Biotechnology Co., Ltd, China).

### Quantification and statistical analysis

The experimental data were statistically analyzed using GraphPad Prism 8.0 software. Quantitative data were presented as means ± SEM. Unpaired *t* test was used to compare means between two groups. One-way ANOVA was used for comparisons between multiple groups. Pearson’s correlation analysis Statistical significance is indicated as follows: *∗∗∗∗p* < 0.0001, *∗∗∗p* < 0.001, *∗∗p* < 0.01, *∗p* < 0.05, *ns* = no significant difference.
