# Supplementary material for: USP25 deubiquitinates cytosolic METTL3 to impede glioma proliferation via an m6A-independent pathway
Source: iScience. 2025 Oct 31;28(12):113918. doi: 10.1016/j.isci.2025.113918 (PMC12661985; doi:10.1016/j.isci.2025.113918)
Supplement: Document S1. Figures S1–S5 and Tables S1 and S2 [file mmc1.pdf]

iScience, Volume *28*

Supplemental information

USP25 deubiquitinates cytosolic METTL3 to impede glioma proliferation via an m6A-independent pathway

Bingchang Zhang, Wanhong Han, Xin Gao, Wenhua Li, Jianhua Yu, Wujie Zhao, Jiawei He, Xiansheng Qiu, Zhenwei Lu, Liwei Zhou, Yahui Hu, Yuanyuan Xie, Yanyan Geng, Hanwen Lu, Wentao Zhao, Xinwen Liao, Shouren Chen, Xiyao Liu, Sifang Chen, Guowei Tan, Yaya Zhang, and Zhanxiang Wang

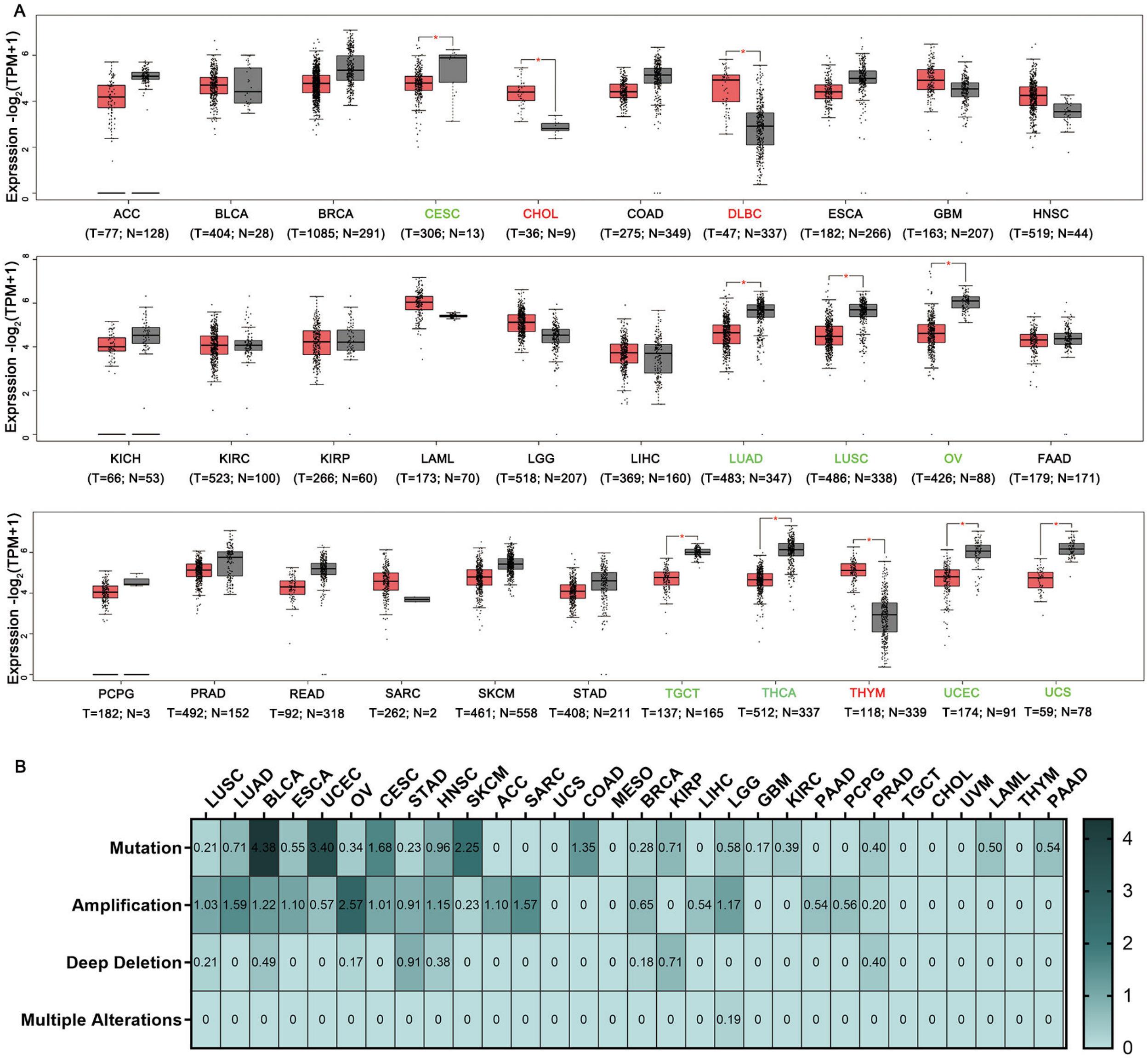

Figure S2. The GEPIA2 dataset and The Cancer Genome Atlas (TCGA) database of METTL3, Related to Figure 2 and STAR Methods.

(A) Comparison of METTL3 mRNA levels between tumor tissues (red) and normal tissues (gray) using GEPIA2 database.

(B) TCGA DNA sequencing results showing the frequencies of METTL3 genomic alterations in the indicatedtumor samples (*n* = 10,888).

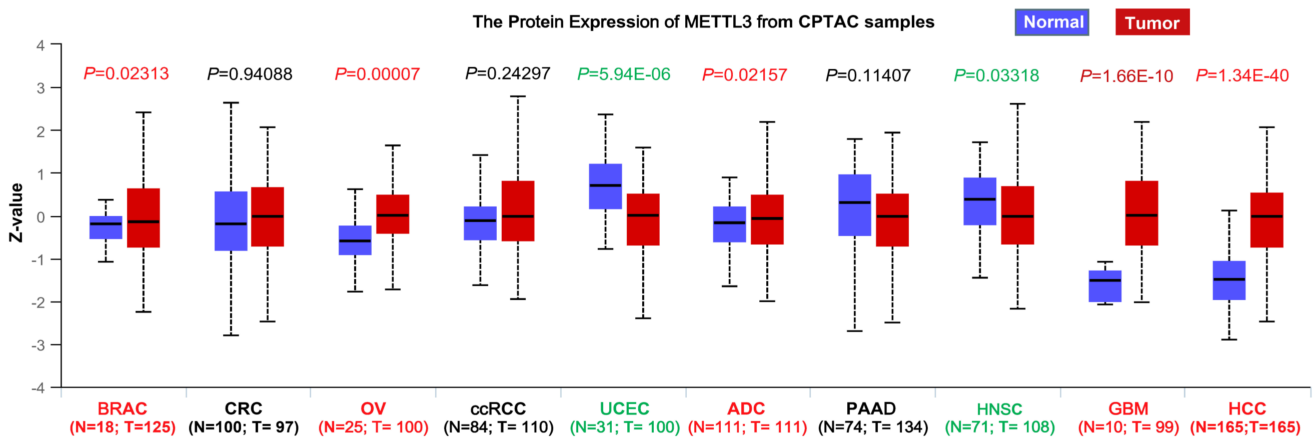

Figure S1. The CPTAC dataset database of METTL3, Related to Figure 1 and STAR Methods.

Comparison of METTL3 protein levels between tumor tissues (red) and normal tissues (blue) using CPTAC database.

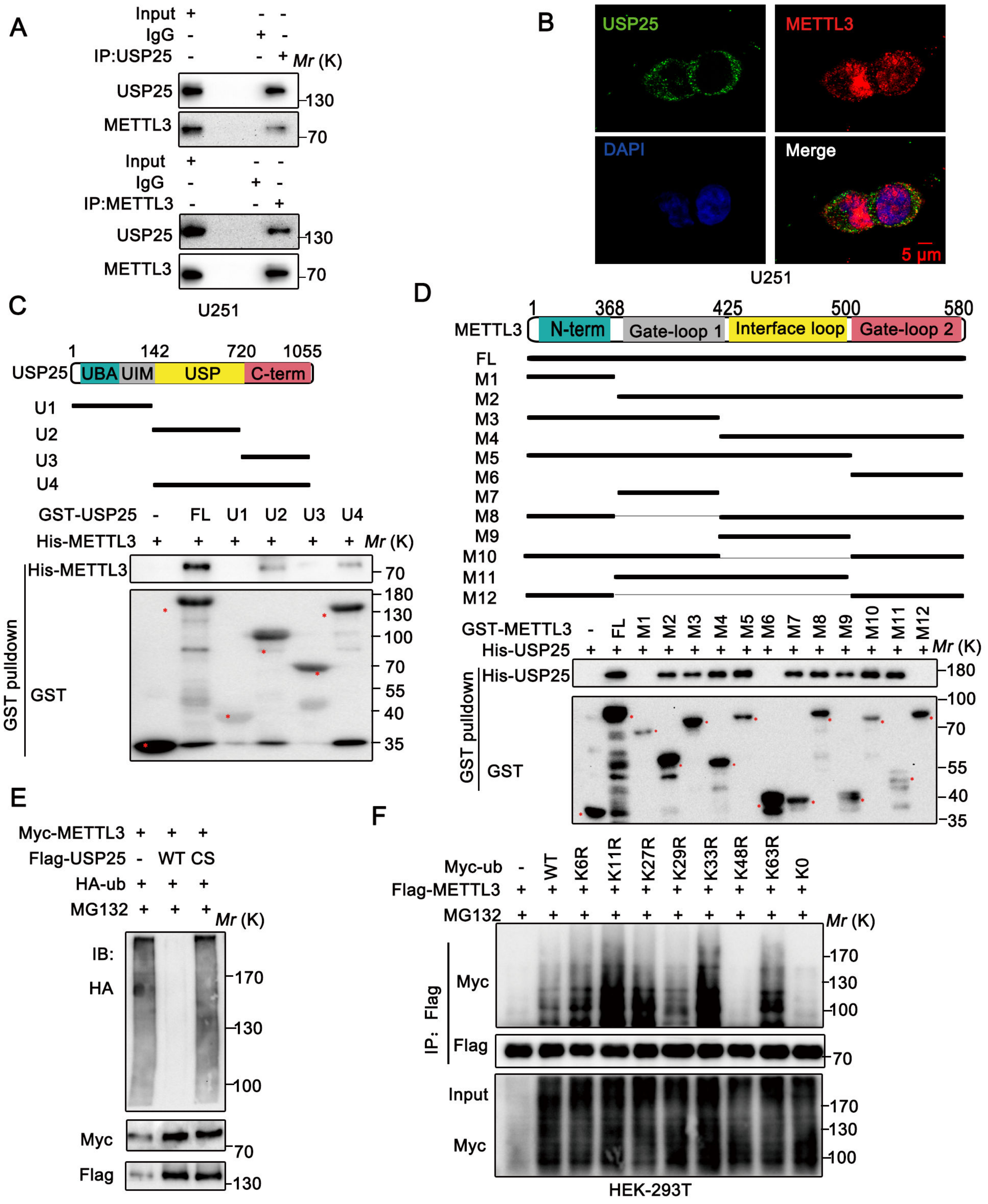
A
Input + - -
IgG - + -
IP:USP25 - - +
Mr (K)
USP25
130
METTL3
70
Input + - -
IgG - + -
IP:METTL3 - - +
USP25
130
METTL3
70
U251
B
USP25
METTL3
DAPI
Merge
5 μm
U251
C
1 142 720 1055
USP25 UBA UIM USP C-term
U1
U2
U3
U4
GST-USP25 - FL U1 U2 U3 U4
His-METTL3 + + + + + +
Mr (K)
His-METTL3
70
180
130
100
70
55
40
35
GST pulldown
GST
D
1 368 425 500 580
METTL3 N-term Gate-loop 1 Interface loop Gate-loop 2
FL
M1
M2
M3
M4
M5
M6
M7
M8
M9
M10
M11
M12
GST-METTL3 - FL M1 M2 M3 M4 M5 M6 M7 M8 M9 M10 M11 M12
His-USP25 + + + + + + + + + + + + + +
Mr (K)
His-USP25
180
100
70
55
40
35
GST pulldown
GST
E
Myc-METTL3 + + +
Flag-USP25 - WT CS
HA-ub + + +
MG132 + + +
Mr (K)
IB:
HA
170
130
100
Myc
70
Flag
130
F
Myc-ub - WT K6R K11R K27R K29R K33R K48R K63R K0
Flag-METTL3 + + + + + + + + + +
MG132 + + + + + + + + + +
Mr (K)
IP: Flag
Myc
170
130
100
Flag
70
Input
Myc
170
130
100
HEK-293T

Figure S3. USP25 binds to METTL3 and regulates its ubiquitination, Related to Figure 2 and STAR Methods.

(A) Endogenous METTL3 and USP25 in U251 cells were captured by IP using anti-METTL3 or anti-USP25 antibody, respectively.

(B) Confocal laser scanning microscopy was used to observe the co-localization of METTL3 (red) and USP25 (green) in U251 cells. Nuclei were visualized with DAPI (blue). Scale bars = 5 μm.

(C) Schematic diagram showing the structure of USP25 and the deletion constructs used (top panel). Purified His-METTL3 was incubated with full-length (FL) or deletion mutants of GST-USP25 coupled to GSH-Sepharose. The proteins retained on Sepharose were then blotted with the indicated antibodies.

(D) Schematic diagram showing the structure of METTL3 and the deletion constructs used (top panel). Purified His-USP25 was incubated with full-length (FL) or deletion mutants of GST-METTL3 coupled to GSH-Sepharose. The proteins retained on Sepharose were then blotted with the indicated antibodies.

(E) HEK-293T cells were transfected with Myc-METTL3 and HA-ubiquitin (HA-ub) and treated with MG-132 for 6 h. Cell lysates were immunoprecipitated with Myc affinity gel, eluted with Myc peptide, and then incubated with purified Flag-USP25^{WT} or Flag-USP25^{C178S} mutant in a cell-free condition. The polyubiquitylated METTL3 protein was detected by anti-HA antibody.

(F) HEK-293T cells were co-transfected with Myc-tagged wild-type (WT) and the indicated KR ubiquitin mutants together with Flag-METTL3 and treated with MG-132 for 6 h. Cell lysates were boiled, immunoprecipitated with Flag affinity gel, and the polyubiquitylated METTL3 protein was detected by anti-Myc antibody.

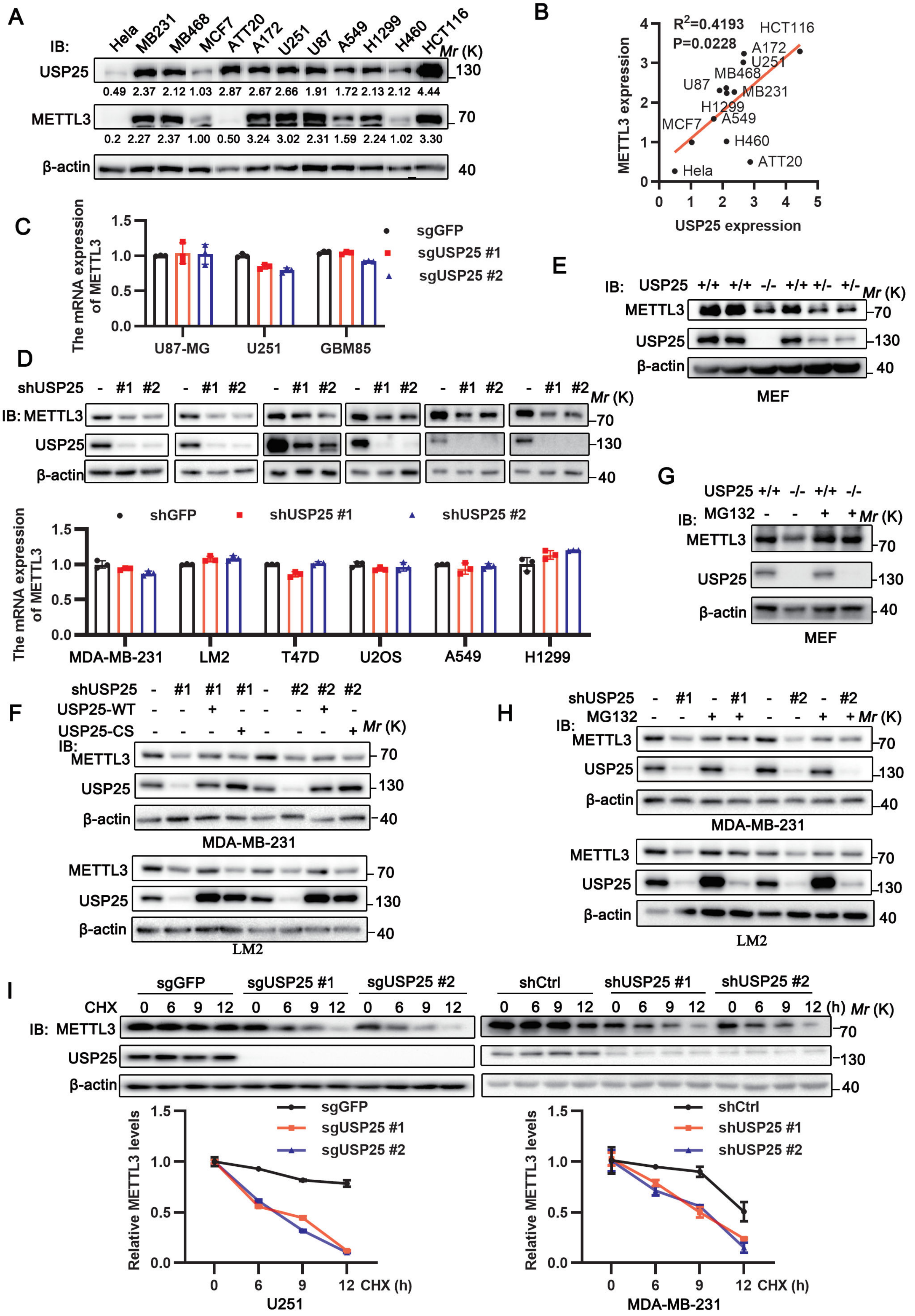
A
IB:
Hela MB231 MB468 MCF7 ATT20 A172 U251 U87 A549 H1299 H460 HCT116
Mr (K)
USP25
130
0.49 2.37 2.12 1.03 2.87 2.67 2.66 1.91 1.72 2.13 2.12 4.44
METTL3
70
0.2 2.27 2.37 1.00 0.50 3.24 3.02 2.31 1.59 2.24 1.02 3.30
β-actin
40
B
R2=0.4193
P=0.0228
HCT116
A172
U251
MB468
U87
MB231
H1299
A549
MCF7
H460
ATT20
Hela
METTL3 expression
USP25 expression
C
The mRNA expression of METTL3
sgGFP
sgUSP25 #1
sgUSP25 #2
U87-MG
U251
GBM85
D
shUSP25 - #1 #2
IB: METTL3
USP25
β-actin
Mr (K)
70
130
40
shGFP
shUSP25 #1
shUSP25 #2
The mRNA expression of METTL3
MDA-MB-231
LM2
T47D
U2OS
A549
H1299
E
IB: USP25 +/+ +/+ -/- +/+ +/- +/-
Mr (K)
METTL3
USP25
β-actin
MEF
F
shUSP25 - #1 #1 #1 - #2 #2 #2
USP25-WT - - + - - - + -
USP25-CS - - - + - - - +
Mr (K)
IB: METTL3
USP25
β-actin
MDA-MB-231
METTL3
USP25
β-actin
LM2
G
USP25 +/+ -/- +/+ -/-
IB: MG132 - - + +
Mr (K)
METTL3
USP25
β-actin
MEF
H
shUSP25 - #1 - #1 - #2 - #2
MG132 - - + + - - + +
Mr (K)
IB: METTL3
USP25
β-actin
MDA-MB-231
METTL3
USP25
β-actin
LM2
I
sgGFP
sgUSP25 #1
sgUSP25 #2
shCtrl
shUSP25 #1
shUSP25 #2
CHX 0 6 9 12 0 6 9 12 0 6 9 12 0 6 9 12 0 6 9 12 0 6 9 12 (h)
Mr (K)
IB: METTL3
USP25
β-actin
Relative METTL3 levels
CHX (h)
U251
MDA-MB-231

Figure S4. USP25 stabilized METTL3, Related to Figure 3 and STAR Methods.
(A and B) Protein expression of METTL3 and USP25 in a broader panel of cancer cell lines were analyzedby Western blot (A), with Pearson's correlation analysis of METTL3 and USP25 protein levels (B).

(C) mRNA levels of METTL3 in broader cancer cell lines infected with control or two individual USP25 sgRNAs were analyzed by qPCR.

(D) Protein expression of METTL3 and USP25, or mRNA levels of METTL3, in broader cancer cell lines infected with control or two individual USP25 shRNAs.

(E) Protein expression of METTL3 and USP25 in $USP25^{-/-}$, $USP25^{+/-}$, or $USP25^{+/+}$ MEFs.

(F) Protein expression of METTL3 and USP25 in MDA-MB-231 cells infected with control or two individual USP25 shRNAs, or USP25 shRNA together with shRNA-resistant $USP25^{WT}$ or $USP25^{C178S}$.

(G) Protein expression of METTL3 and USP25 in $USP25^{-/-}$ or $USP25^{+/+}$ MEF cells treated with or without 25 µM MG132 for 6 h was analyzed by Western blot.

(H) Protein expression of METTL3 and USP25 in MDA-MB-231 LM2 cells infected with control or two individual USP25 shRNAs, and treated with or without 25 µM MG132 for 6 h, analyzed by Western blot.

(I) Protein expression of METTL3 and USP25 in U251 cells infected with control or two individual USP25 sgRNAs and treated with or without 50 µg/µL cycloheximide (CHX). Western blot analysis was performed, and the intensity of METTL3 was normalized to β-actin (loading control) at each time point, with values normalized to the 0 h time point.

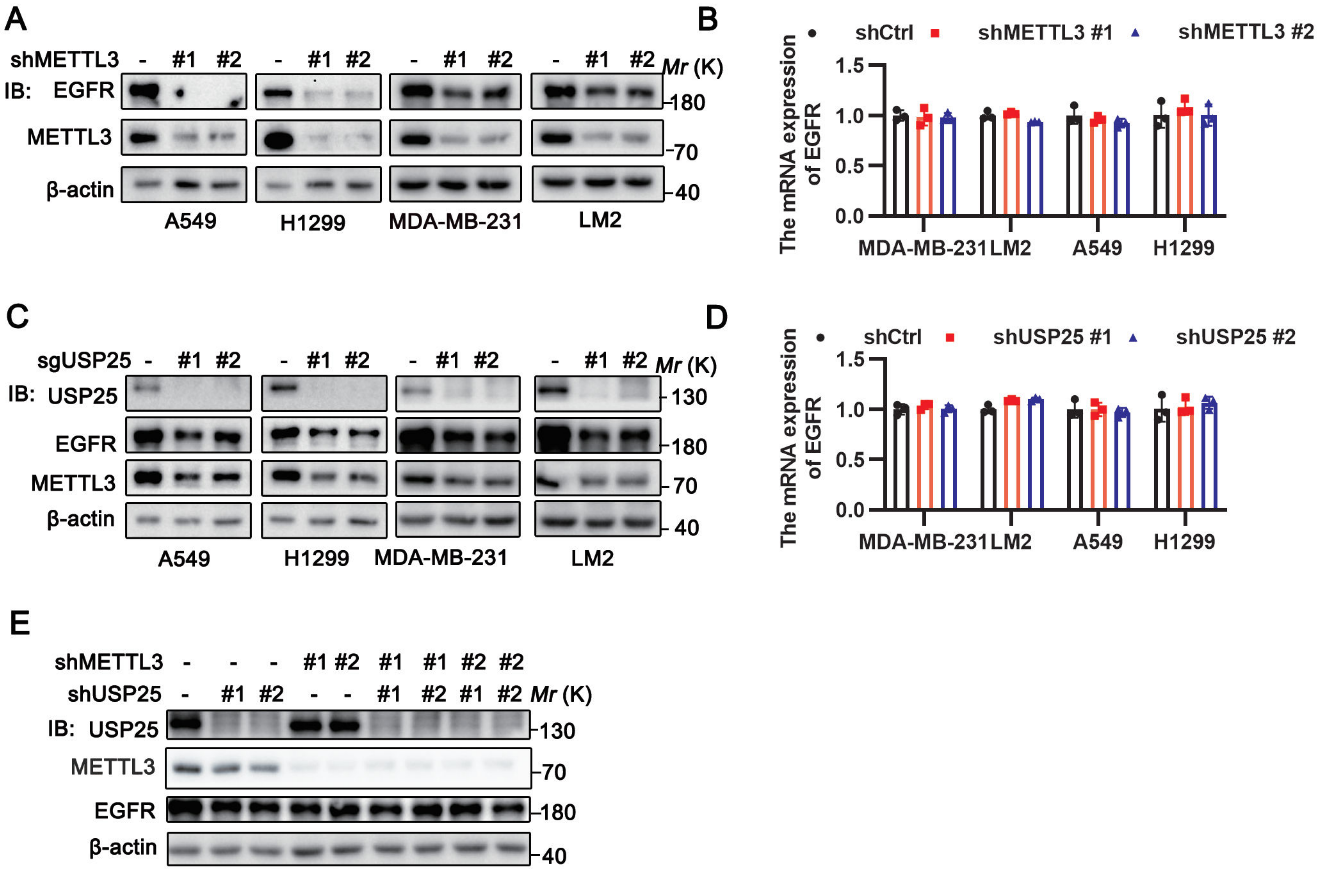

Figure S5. USP25 promotes EGFR expression through cytoplasmic METTL3, Related to Figure 5 and STAR Methods.

(A) Protein expression of EGFR in A549, H1299, MDA-MB-231 and LM2 cells infected with control or two individual METTL3 shRNAs was analyzed by Western blot.

(B) mRNA levels of EGFR in A549, H1299, MDA-MB-231 and LM2 cells infected with control or two individual METTL3 shRNAs was analyzed by qPCR.

(C) Protein expression of EGFR in A549, H1299, MDA-MB-231 and LM2 cells infected with control or two individual USP25 shRNAs was analyzed by Western blot.

(D) mRNA levels of EGFR in A549, H1299, MDA-MB-231 and LM2 cells infected with control or two individual USP25 shRNAs was analyzed by qPCR.

(E) In MDA-MB-231 cells, individual or combined knockdown of USP25 and METTL3 was performed, and the protein expression levels of EGFR was analyzed by Western blot.

Table S1 The Clinical information of TMA Patient, Related to Figure 1 and STAR Methods.

Patient	Gender	Age	Grade	Survival status	5-year OS_ days	IDH1 mutation	ATRX mutation	TP53 mutation	H3K27M	TERT promoter mutation	MGMT promoter methylation	EGFR amplification	CDKN2A/B homozygous deletion	1p/19q codeletion
1	Male	42	II	NA	NA	+	+	+	-	-	+	-	-	-
2	Male	40	II	NA	NA	-	+	-	-	-	+	-	-	+
3	Male	52	II	NA	NA	+	-	+	-	-	-	-	-	-
4	Male	58	II	NA	NA	+	-	-	+	-	+	-	-	-
5	Male	56	Ⅲ	NA	NA	+	+	-	-	+	-	-	-	+
6	Male	65	Ⅲ	NA	NA	-	+	-	-	-	-	-	-	-
7	Female	46	Ⅲ	NA	NA	-	-	+	-	-	+	+	-	-
8	Male	46	Ⅲ	NA	NA	+	-	-	-	-	-	-	-	+
9	Male	61	Ⅲ	NA	NA	-	-	+	-	-	-	-	-	-
10	Female	40	Ⅲ	NA	NA	+	-	+	+	-	-	+	-	-
11	Male	6	Ⅲ	NA	NA	-	+	+	-	-	+	-	-	-
12	Female	58	Ⅳ	NA	NA	-	-	-	-	+	-	-	+	-
13	Male	67	Ⅳ	NA	NA	-	+	-	+	-	-	-	-	-
14	Female	12	Ⅳ	NA	NA	-	+	-	+	-	-	-	-	-
15	Female	56	Ⅳ	NA	NA	-	+	-	+	-	+	-	-	-
16	Female	38	Ⅳ	NA	NA	+	-	+	-	-	-	-	+	-
17	Female	44	Ⅳ	NA	NA	-	+	-	-	-	-	+	-	-
18	Male	7	Ⅳ	NA	NA	-	+	-	+	-	-	-	-	-
19	Female	56	Ⅳ	NA	NA	-	-	-	-	-	-	-	-	-
20	Female	50	Ⅳ	NA	NA	-	-	+	+	-	-	-	-	-
21	Male	59	Ⅳ	NA	NA	-	-	+	-	-	-	+	-	-

22	Female	50	IV	NA	NA	-	-	-	-	+	-	+	-	-
23	Female	77	IV	NA	NA	-	+	+	-	-	-	+	-	-
24	Male	65	IV	NA	NA	-	+	+	-	-	-	-	-	-
25	Male	15	IV	NA	NA	-	+	+	+	-	-	-	-	-
26	Female	61	IV	NA	NA	+	-	+	-	-	-	-	-	-
27	Male	59	IV	NA	NA	-	-	-	-	+	-	-	-	-
28	Female	56	IV	NA	NA	-	+	+	-	-	-	-	-	-
29	Male	43	IV	NA	NA	+	-	+	-	-	-	-	-	-
30	Female	38	IV	NA	NA	-	-	-	-	-	-	-	-	-
31	Female	31	IV	NA	NA	-	-	+	-	-	-	-	-	-
32	Female	80	IV	NA	NA	-	-	-	-	-	-	-	-	-
33	Male	74	IV	NA	NA	-	+	+	+	+	-	-	-	-
34	Male	69	IV	NA	NA	-	+	+	-	-	-	-	-	-
35	Female	51	IV	NA	NA	-	+	+	+	-	-	-	-	-
36	Female	54	IV	NA	NA	-	+	-	+	+	-	+	+	-
37	Male	52	IV	NA	NA	-	+	-	+	+	-	+	+	-
38	Male	66	IV	NA	NA	-	+	+	-	+	+	-	-	-
39	Female	39	normal	NA	NA	NA	NA	NA	NA	NA	NA	NA	NA	NA
40	Male	49	normal	NA	NA	NA	NA	NA	NA	NA	NA	NA	NA	NA
41	Female	54	normal	NA	NA	NA	NA	NA	NA	NA	NA	NA	NA	NA
42	Male	60	normal	NA	NA	NA	NA	NA	NA	NA	NA	NA	NA	NA
43	Male	20	normal	NA	NA	NA	NA	NA	NA	NA	NA	NA	NA	NA
44	Female	75	normal	NA	NA	NA	NA	NA	NA	NA	NA	NA	NA	NA

Table S2 The Clinical information of tissue for Western blot analysis, Related to Figure 3 and STAR Methods.

Patient	Gender	Age	Grade	Survival status	5-year OS_ days	IDH1 mutation	ATRX mutation	TP53 mutation	H3K27M	TERT promoter mutation	MGMT promoter methylation	EGFR amplification	CDKN2A/B homozygous deletion	1p/19q codeletion
1	Male	42	II	NA	NA	+	+	+	-	-	+	-	-	-
2	Male	40	II	NA	NA	-	+	-	-	-	+	-	-	+
3	Male	52	II	NA	NA	+	-	+	-	-	-	-	-	-
4	Male	58	II	NA	NA	+	-	-	+	-	+	-	-	-
5	Male	56	Ⅲ	NA	NA	+	+	-	-	+	-	-	-	+
6	Male	65	Ⅲ	NA	NA	-	+	-	-	-	-	-	-	-
7	Female	46	Ⅲ	NA	NA	-	-	+	-	-	+	+	-	-
8	Male	46	Ⅲ	NA	NA	+	-	-	-	-	-	-	-	+
9	Male	61	Ⅲ	NA	NA	-	-	+	-	-	-	-	-	-
10	Female	40	Ⅲ	NA	NA	+	-	+	+	-	-	+	-	-
11	Male	6	Ⅲ	NA	NA	-	+	+	-	-	+	-	-	-
12	Female	58	IV	NA	NA	-	-	-	-	+	-	-	+	-
13	Male	67	IV	NA	NA	-	+	-	+	-	-	-	-	-
14	Female	12	IV	NA	NA	-	+	-	+	-	-	-	-	-
15	Female	56	IV	NA	NA	-	+	-	+	-	+	-	-	-
16	Female	38	IV	NA	NA	+	-	+	-	-	-	-	+	-
17	Female	44	IV	NA	NA	-	+	-	-	-	-	+	-	-
18	Male	7	IV	NA	NA	-	+	-	+	-	-	-	-	-
19	Female	56	IV	NA	NA	-	-	-	-	-	-	-	-	-
20	Female	50	IV	NA	NA	-	-	+	+	-	-	-	-	-
21	Male	59	IV	NA	NA	-	-	+	-	-	-	+	-	-
22	Female	50	IV	NA	NA	-	-	-	-	+	-	+	-	-

23	Female	77	Ⅳ	NA	NA	-	+	+	-	-	-	+	-	-
24	Male	65	Ⅳ	NA	NA	-	+	+	-	-	-	-	-	-
25	Male	15	Ⅳ	NA	NA	-	+	+	+	-	-	-	-	-
26	Female	61	Ⅳ	NA	NA	+	-	+	-	-	-	-	-	-
27	Male	59	Ⅳ	NA	NA	-	-	-	-	+	-	-	-	-
28	Female	56	Ⅳ	NA	NA	-	+	+	-	-	-	-	-	-
29	Male	43	Ⅳ	NA	NA	+	-	+	-	-	-	-	-	-
30	Female	38	Ⅳ	NA	NA	-	-	-	-	-	-	-	-	-
31	Female	31	Ⅳ	NA	NA	-	-	+	-	-	-	-	-	-
39	Female	39	normal	NA	NA	NA	NA	NA	NA	NA	NA	NA	NA	NA
40	Male	49	normal	NA	NA	NA	NA	NA	NA	NA	NA	NA	NA	NA
41	Female	54	normal	NA	NA	NA	NA	NA	NA	NA	NA	NA	NA	NA
42	Male	60	normal	NA	NA	NA	NA	NA	NA	NA	NA	NA	NA	NA
43	Male	20	normal	NA	NA	NA	NA	NA	NA	NA	NA	NA	NA	NA
44	Female	75	normal	NA	NA	NA	NA	NA	NA	NA	NA	NA	NA	NA
45	Female	45	normal	NA	NA	NA	NA	NA	NA	NA	NA	NA	NA	NA
46	Male	4	normal	NA	NA	NA	NA	NA	NA	NA	NA	NA	NA	NA
47	Female	39	normal	NA	NA	NA	NA	NA	NA	NA	NA	NA	NA	NA
48	Male	48	normal	NA	NA	NA	NA	NA	NA	NA	NA	NA	NA	NA
49	Male	55	normal	NA	NA	NA	NA	NA	NA	NA	NA	NA	NA	NA
50	Female	59	I	NA	NA	-	-	-	-	-	-	-	-	-
51	Male	33	I	NA	NA	-	-	-	-	-	-	-	-	-
52	Female	46	Ⅲ	NA	NA	-	-	+	-	-	+	-	-	-
53	Male	46	Ⅲ	NA	NA	+	-	-	-	-	-	-	-	-
54	Female	48	Ⅲ	NA	NA	+	+	+	-	-	+	-	-	-
55	Male	65	Ⅲ	NA	NA	+	+	-	-	-	-	-	-	-
